# Selective neurodegeneration of the hippocampus caused by chronic cerebral hypoperfusion: F-18 FDG PET study in rats

**DOI:** 10.1371/journal.pone.0262224

**Published:** 2022-02-10

**Authors:** Jung-In Lee, Ji Sun Lim, Jeong-Ho Hong, Shin Kim, Sang-Woo Lee, Hyun Dong Ji, Kyoung Sook Won, Bong-Il Song, Hae Won Kim

**Affiliations:** 1 Department of Nuclear Medicine, Keimyung University Dongsan Hospital, Daegu, Republic of Korea; 2 Department of Neurology, Keimyung University Dongsan Hospital, Daegu, Republic of Korea; 3 Department of Immunology, Keimyung University School of Medicine, Daegu, Republic of Korea; 4 Department of Nuclear Medicine, School of Medicine, Kyungpook National University, Daegu, Republic of Korea; 5 Department of Nuclear Medicine, School of Medicine & Institute for Medical Science, Keimyung University, Daegu, Korea; Albany Medical College, UNITED STATES

## Abstract

**Background:**

Chronic cerebral hypoperfusion (CCH) is known to induce Alzheimer’s disease (AD) pathology, but its mechanism remains unclear. The purpose of this study was to identify the cerebral regions that are affected by CCH, and to evaluate the development of AD pathology in a rat model of CCH.

**Methods:**

A rat model of CCH was established by bilaterally ligating the common carotid arteries in adult male rats (CCH group). The identical operations were performed on sham rats without arteries ligation (control group). Regional cerebral glucose metabolism was evaluated at 1 and 3 months after bilateral CCA ligation using positron emission tomography with F-18 fluorodeoxyglucose. The expression levels of amyloid β40 (Aβ40), amyloid β42 (Aβ42), and hyperphosphorylated tau were evaluated using western blots at 3 months after the ligation. Cognitive function was evaluated using the Y-maze test at 3 months after the ligation.

**Results:**

At 1 month after the ligation, cerebral glucose metabolism in the entorhinal, frontal association, motor, and somatosensory cortices were significantly decreased in the CCH group compared with those in the control group. At 3 months after the ligation, cerebral glucose metabolism was normalized in all regions except for the anterodorsal hippocampus, which was significantly decreased compared with that of the control group. The expression of Aβ42 and the Aβ42/40 ratio were significantly higher in the CCH group than those in the control group. The phosphorylated-tau levels of the hippocampus in the CCH group were significantly lower than those in the control group. Cognitive function was more impaired in the CCH group than that in the control group.

**Conclusion:**

Our findings suggest that CCH causes selective neurodegeneration of the anterodorsal hippocampus, which may be a trigger point for the development of AD pathology.

## Introduction

Alzheimer’s disease (AD) accounts for 60% to 70% of cases of dementia, which according to current estimates, will affect up to 81.1 million patients worldwide by 2040 [1]. Deposition of amyloid beta (Aβ) and neurofibrillary tangles are the main pathological hallmarks of AD, which are respectively caused by aggregation of Aβ peptides and hyperphosphorylated tau (p-tau) in the brain [2, 3]. Although the cause of AD pathology has not been clearly identified, previous studies have reported the relationship between cerebral hypoperfusion and AD pathology [4]; therefore, cerebral hypoperfusion may be a predisposing factor in the pathological progression of AD. This is supported by the fact that vascular risk factors that cause cerebral hypoperfusion, such as hypertension, diabetes, hypercholesterolemia, and smoking, are also risk factors of AD [5]. In addition, cerebral hypoperfusion is known to potentiate other processes associated with AD such as mitochondrial failure, oxidative stress, and neuroinflammation [6, 7].

Cerebral hypoperfusion can occur in two patterns, acute or chronic, depending on the rate at which cerebral vessels narrow; both cause cognitive deficits in various degrees [8]. While acute cerebral hypoperfusion leads to an infarction within approximately 3 h via necrosis of neuronal cells [9], chronic cerebral hypoperfusion (CCH) causes neurodegeneration over a period of months to years through neuronal apoptosis without infarction [10]. The important role of CCH in AD has already emerged at the vanguard of neurology research [11]. Recent in vivo studies have revealed that CCH accelerates AD pathology, including Aβ aggregation and cognitive dysfunction [12, 13]. In turn, Aβ aggregation has been shown to exacerbate inefficient microcirculation and cause blood brain barrier disruption, indicating a vicious cycle between CCH and AD pathology [14]. However, it is not clear which cerebral regions are affected by CCH to cause AD pathology.

Positron emission tomography (PET) with F-18 fluorodeoxyglucose (FDG) is a minimally invasive diagnostic brain imaging procedure that is used to assess regional cerebral glucose metabolism. F-18 FDG uptake on PET reflects regional glucose consumption and synaptic function in the brain [15, 16]. It has been reported that decreased glucose metabolism in specific cerebral regions is an important indicator for the detection of early AD [17]. In a previous study, F-18 FDG PET of a CCH rat model revealed that CCH results in decreased glucose metabolism in the hippocampus, suggesting that CCH can induce AD pathology [18]. However, the previous study did not provide results on cerebral glucose metabolism during the early stage of CCH, and it could not be established whether the decreased glucose metabolism in the hippocampus was due to an acute change similar to vascular dementia or a chronic change similar to AD development. In addition, since the previous study assessed cerebral blood flow (CBF) for up to 1 month after CCH, it was difficult to determine how the hippocampus was affected by the improvement in CBF after 3 months. Since serial evaluations of regional cerebral glucose metabolism with F-18 FDG PET and serial measurements of CBF can reveal sequential changes in cerebral glucose metabolism by CCH, they may facilitate a better understanding of the pathophysiology of CCH during AD development. Thus, the purpose of this study was to identify the cerebral regions that are affected by CCH using F-18 FDG PET, and to evaluate the development of AD pathology in a rat model of CCH.

## Materials and methods

### Animals

**Fig 1:**
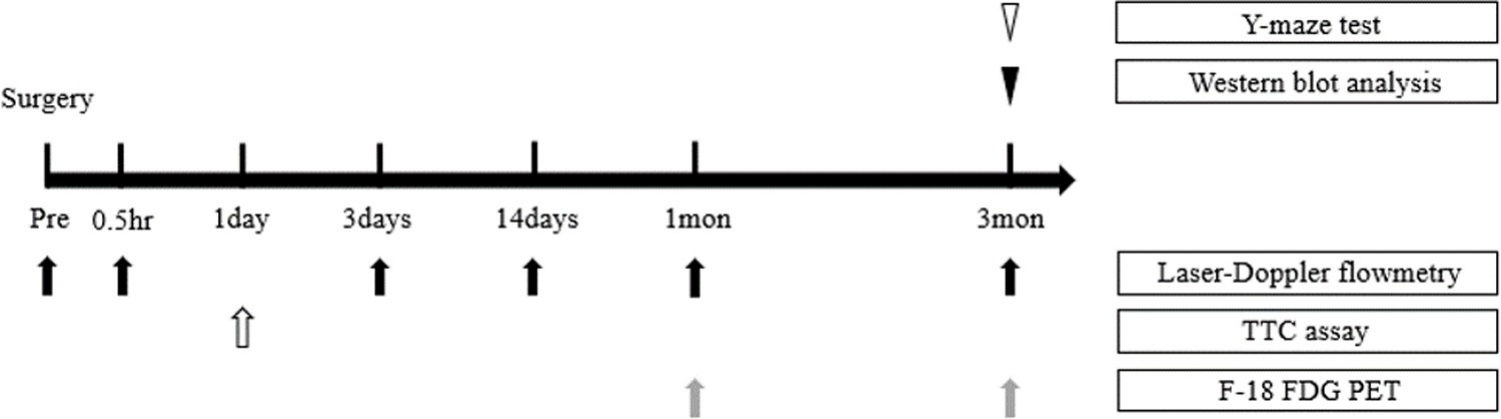
Experimental design. CCH and control groups were performed in 11-week-old Wistar rats. To confirm that there was no cerebral infarction after surgery, TTC assay performed on 7 rats in each group randomly, immediately after surgery. F-18 FDG PET imaging was performed 1 and 3 months after surgery. The Y-maze test and western blot analysis were performed 3 months after surgery. CCH: chronic cerebral hypoperfusion; TTC: 2,3,5-triphenyltetrazolium chloride; F-18 FDG: F-18 fluorodeoxyglucose; PET: positron emission tomography.

Eight-week-old male Wistar rats (250–300 g body weight) purchased from Central Lab Animal Korea, Inc. were used for this study. The rats were kept in standard cages with 12 h light/12 h dark cycles (8:00 lights on) and humidity that is kept under control at a temperature of 22–24°C (55–60 percent). Food and drink were available at all times. The surgery, CBF, 2,3,5-triphenyltetrazolium chloride (TTC) assay, F-18 FDG PET, western blot analysis, and Y-maze tests were performed according to the schedule illustrated in Fig 1. All experiments in this study were performed in accordance with the guidelines for animal research from the National Institutes of Health. The Keimyung University Institutional Ethics Committee (Daegu, Korea) approved all the animal experiments (KM-2019-13R1).

### Bilateral common carotid artery ligation surgery

The rat model of CCH was established as previously described [19]. In 22 Wistar rats, the bilateral common carotid artery (CCA) was doubly ligated (CCH group), whereas 22 sham-operated rats were treated to the same procedures without CCA ligation (control group). The rats were sedated with 4.0% isoflurane in N_2_O/O_2_ (70:30) and maintained with 2.0% isoflurane in N_2_O/O_2_ (70:30). (70:30). Throughout the duration of the process, the core temperature was maintained between 37 and 38°C. To enable the separation of the bilateral CCA from the surrounding tissues, a tiny incision was carefully made. The CCA stump was wrapped with polylysine-coated nylon (Nylon Monofilament Suture, Fine Science Tools Inc., Foster City, USA). Only anesthesia and vascular dissection were performed in the control group. Rats were separated into cages for post-surgery care.

### Laser-Doppler flowmetry

The CBF was measured in 15 rats in each group (CCH and control) using laser-Doppler flowmetry (OMEGA FLOW FLO-C1 BV, OMEGAWABE, Tokyo, Japan), as previously reported [19, 20]. An incision was made with surgical scissors, and the connective tissue was scraped off to expose the skull under deep anesthesia with 4.0% isoflurane in N_2_O/O_2_ (70:30). To position the laser-Doppler flowmetry (LDF) probe within the same area of the skull for longitudinal measurements, a ruler was used to identify the area 1-mm posterior and 2.5-mm lateral to the bregma. The (LDF) probe was applied to the skull, and the CBF output was read. If the signal was good, the LDF probe was fixed using dental resin. The dental resin was easily removed from the skull together with the LDF probe, and the residual resin was carefully removed with sandpaper to prevent damage to the skull. After the measurement of CBF, the incision was closed using Vetbond. CBF measurements were taken right before surgery (baseline), as well as 30 minutes, 3 days, 14 days, 1 month, and 3 months later. The CBF ratio (%) was calculated as a percentage of the baseline CBF before the surgery.

### TTC assay

A TTC assay was completed 1 day after bilateral CCA ligation to assess cerebral infarction. A total of seven rats from each group (control and CCH groups) were sacrificed. Their brains were extracted from them and sliced into five 2 mm thick coronal pieces. These sections were submerged in 2% TTC (Sigma-Aldrich, Steinheim, Germany) in saline for 15 minutes before being fixed overnight in 4% paraformaldehyde. TTC showed normal tissue parts red, while white portions of the brain indicated cerebral infarct sites.

### F-18 FDG PET

To evaluate cerebral glucose metabolism, 9 rats from each group (control group and CCH group) underwent an F-18 FDG PET at 1 month after bilateral CCA ligation, and 14 rats from each group underwent an F-18 FDG PET at 3 months using the Triumph II PET/CT system (Lab-PET8; Gamma Medica-Ideas, Waukesha, WI, USA). Before the PET scan, the rats were fasted for 12 hours. They were sedated with 2.0% isoflurane in N_2_O/O_2_ (70:30) and injected with roughly 37 MBq of F-18 FDG into the tail vein. The rats underwent PET scanning approximately 30 min after F-18 FDG injection to acquire whole-brain images; the PET scan lasted for 20 min. The acquired data were assumed to indicate cerebral glucose metabolism. For spatiotemporal quantification of the cerebral glucose metabolism, a volume-of interest (VOI) analysis was performed for each scan with the use of the PMOD software package (PMOD Technologies, Ltd., Zurich, Switzerland) in conjunction with the W. Schiffer rat brain template and atlas, as stated previously [19, 21]. PMOD was used to transform each of the rat brain PET datasets to the appropriate space, and the W. Schiffer VOI brain atlas was automatically applied to measure the F-18 FDG uptake to acquire standardized F-18 FDG uptake values within defined subregions of the rat brain. The W. Schiffer brain VOI atlas was used in an iterative fashion with the standard brain model to further optimize the fusion of the experimental data. The regional standardized F-18 FDG uptake values ratio (SUVR) was calculated by dividing the standardized F-18 FDG uptake value for the individual target region by that for the bilateral cerebellum.

### Western blot analysis

At 3 months following bilateral CCA ligation, Western blots were used to assess the expression of Bax, TNF-α, p-tau (Thr231), soluble Aβ, Aβ40, and Aβ42. T-PERTM Tissue Protein Extraction Reagent (78510; Thermo Fisher Scientific, Waltham, MA, USA) was used to homogenize brain tissue (left hippocampus) from 15 rats in each experimental group, which was then incubated at 4°C for 30 minutes with proteinase inhibitor cocktail tablet 1 (cOmplete Mini, EDTA-free; Roche Applied Science, Germany) and PhosSTOP EASY (Roche Applied Science, Germany). Samples were then centrifuged at 15,000 rpm for 15 min at 4°C. The amount of protein (10 μg) was measured using a bicinchoninic acid assay protein assay (Pierce, Thermo Fisher Scientific, Waltham, MA, USA). Proteins separated using 10% sodium dodecyl sulfate-polyacrylamide gel electrophoresis were transferred to nitrocellulose membranes, and immunoreactive bands were visualized using a chemiluminescent reagent (SuperSignal West Femto Maximum Sensitivity Substrate; Thermo Fisher Scientific, Waltham, MA, USA). The signals of the bands were quantified with the scion image software using a FUSIONSOLO5 (KOREA BIOMICS, Korea). The following antibodies were used: anti-bax (1:1000) (ab182733, Abcam, Cambridge, MA, USA), anti-TNF-α (1:1000) (ab6671, Abcam, Cambridge, MA, USA), anti-hyperphosphorylated tau (1:1000) (ab151559, Abcam, Cambridge, MA, USA), anti- Amyloid β (ab216436, Abcam, Cambridge, MA, USA), anti-Amyloid β1–40 (1:1000) (ab17295, Abcam, Cambridge, MA, USA), anti-Amyloid β1–42 (1:1000) (ab201061, Abcam, Cambridge, MA, USA), anti-GAPDH (1:2000) (2118, Cell Signaling Technology, Danvers, MA, USA), anti-mouse IgG HRP-linked antibody (1:1000) (Santa Cruz Biotechnology, Santa Cruz, CA, USA), and anti-rabbit IgG HRP-linked antibody (1:1000) (Santa Cruz Biotechnology, Santa Cruz, CA, USA).

### Y-maze test

The Y-maze test was performed to assess the spatial working memory of the 7 rats in each experimental group as described previously [22]. The apparatus for the Y-maze comprised three arms (500 mm long, 150 mm high, and 100 mm wide; labeled A, B, and C) diverging at a 120° angle. The Y-maze test was performed in a dimly illuminated testing room 3 months after surgery. Rats moved into the room and allowed them to adapt for 1 h. Each rat was gently placed at the end of start arm and allowed to freely move through the Y-maze for 10 min without reinforcers, such as food, water, or electric shock. During the test, the operator was blinded to the group allocation of each animal and the behaviors of the rats were recorded using a video camera. The floor of the maze was cleaned with 70% ethanol after each rat was tested to avoid olfactory cues. An actual alternation was defined as consecutive entries into all three arms [23]. The maximum number of alternations was then calculated by subtracting two units from the total number of arm entries, and the percentage of spontaneous alternation was calculated as follows: spontaneous alternation (%) = (actual number of alternations/maximum number of alternations) × 100. The total number of arms entered during the test was also recorded.

### Statistical analysis

The results are expressed as the mean ± standard deviation (SD). Statistical Package for the Social Sciences (SPSS) software version 26.0 (IBM corporation, Armonk, NY, USA) was used for the statistical analyses. Differences in CBF; infarct volume; regional SUVR; and the recognition index between the CCH and control groups were evaluated using the Student’s two-tailed t-test. The expression levels for Bax, TNF-α, p-tau, Aβ, Aβ40 and Aβ42 in the CCH group were normalized to the average of the control group and were compared with those of the control group using one-sample t-test. A value of *p* < 0.05 was considered statistically significant.

## Results

### Chronic cerebral hypoperfusion

**Fig 2:**
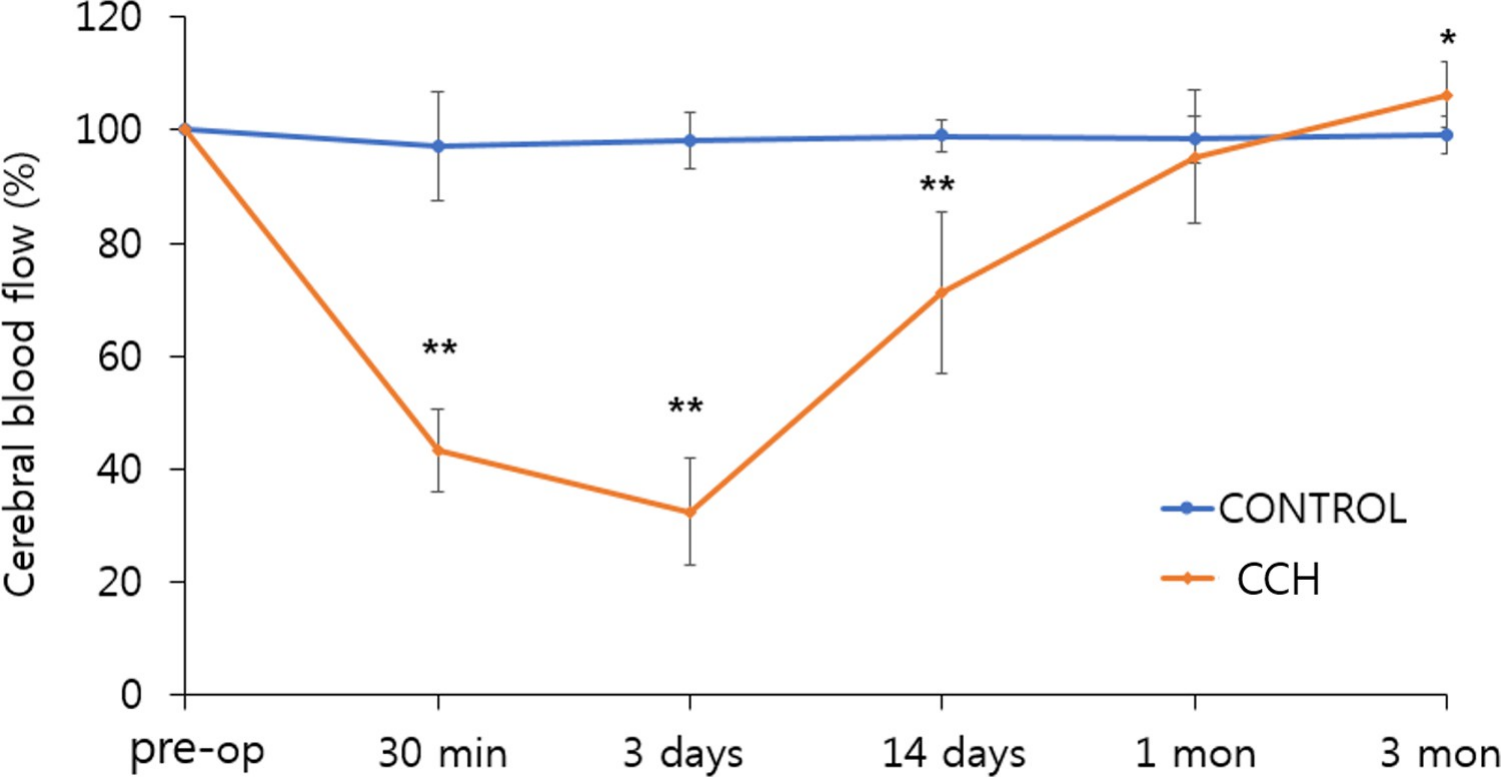
Comparison of CBF between the CCH and control groups. The CBF was measured before and at 30 min, 3 days, 14 days, 1 month, and 3 months after surgery, using laser-Doppler flowmetry. At both 30 min and 3 days after surgery, there was a significant decrease in the CBF ratio in the CCH group compared with that in the control group. At 14 days, the CBF ratio in the CCH group began to recover but remained significantly lower than that in the control group. In contrast, the CBF ratio in the CCH group was slightly but significantly increased compared with that of the control group at 3 months after surgery. Asterisks indicate statistical significance: **p < 0.01 *p < 0.05. CBF: cerebral blood flow; CCH: chronic cerebral hypoperfusion.

Using laser-Doppler flowmetry, the CBF was measured just before and at 30 min, 3 days, 14 days, 1 month, and 3 months after the bilateral CCA ligation (Fig 2). At 30 min and 3 days after the surgery, there was a significant decrease in the CBF ratio in the CCH group compared with that in the control group (43.2 ± 7.3% vs. 97.1 ± 9.6%, *p* < 0.001 and 32.4 ± 9.2% vs. 98.1 ± 5.1%, *p* < 0.001, respectively). At 14 days, the CBF ratio of the CCH group began to recover but remained significantly lower than that of the control group (71.3 ± 14.3% vs. 98.9 ± 2.9%, *p* < 0.001). At 3 months, the CBF ratio of the CCH group was slightly increased compared to that of the control group (106.2 ± 5.8% vs. 99.0 ± 3.4%, *p* = 0.012).

**Fig 3:**
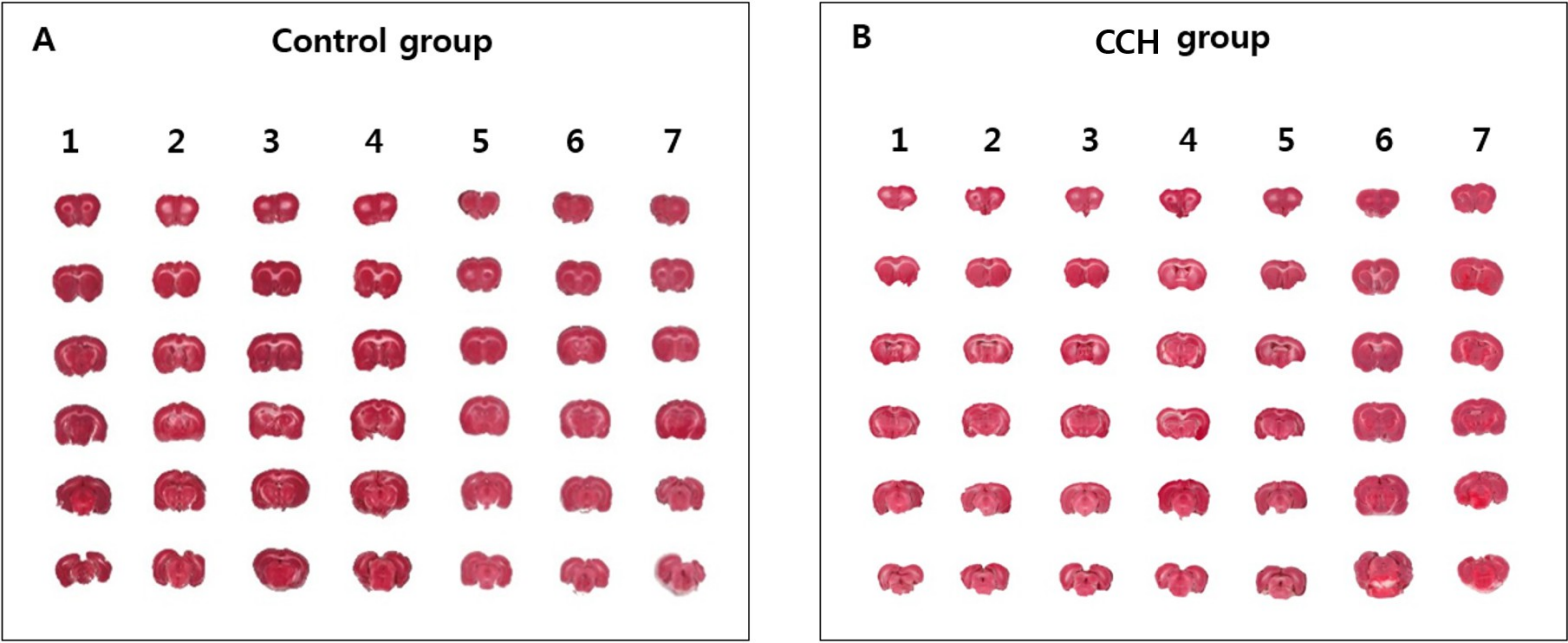
Comparison of cerebral infarct volume between the CCH and control groups. Cerebral infarct volume was evaluated by the TTC assay. (A) TTC assay showing that none of the seven rats in the control group exhibited cerebral infarction. (B) In the CCH group, none of the seven rats exhibited a cerebral infarct. CCH: chronic cerebral hypoperfusion; TTC: 2,3,5-triphenyltetrazolium chloride.

The TTC assay showed that there was no cerebral infarct in the bilateral cerebral hemisphere in both the control and CCH groups (Fig 3).

### Cerebral glucose metabolism

**Fig 4:**
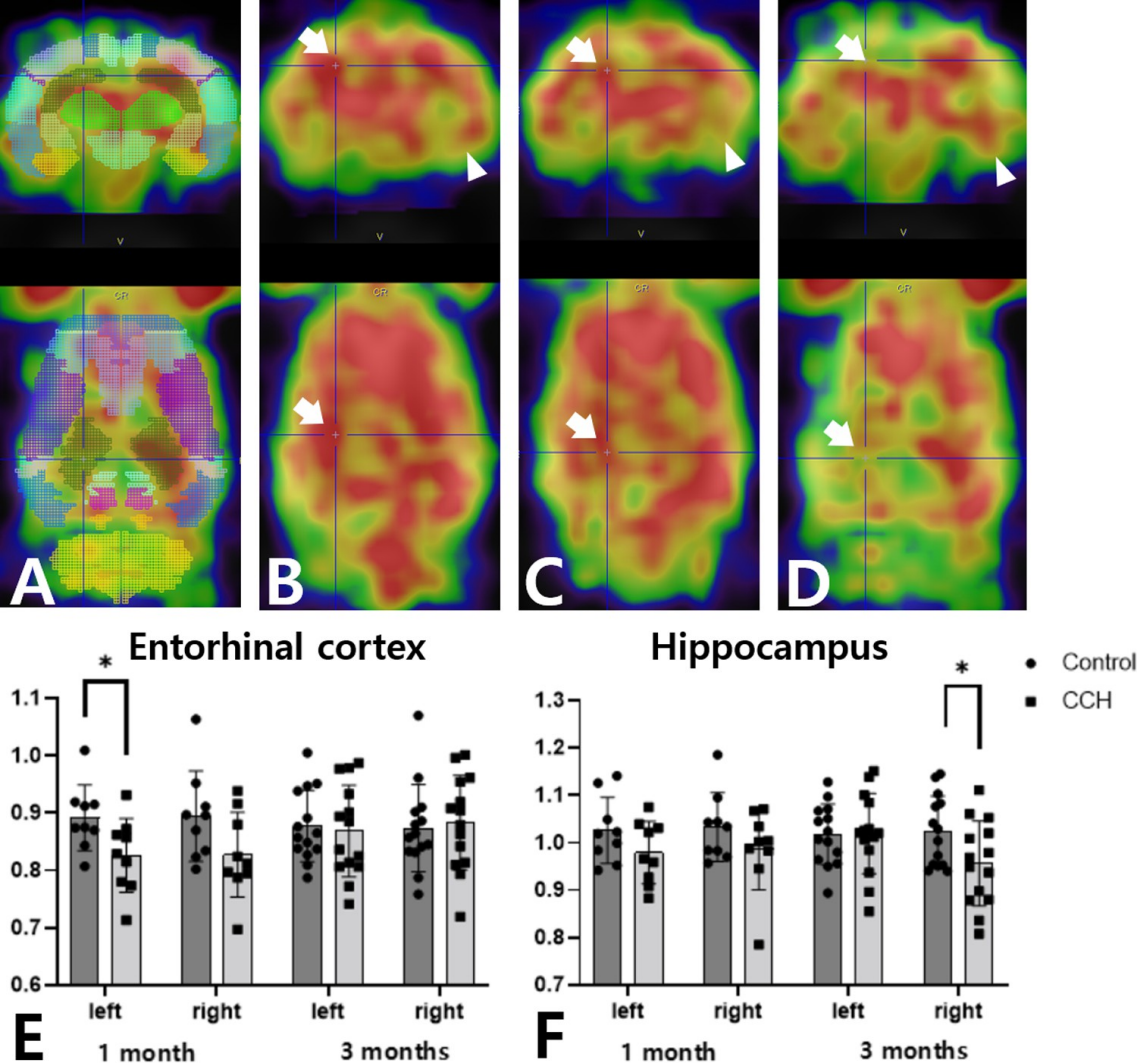
Cerebral glucose metabolism measured by F-18 FDG PET. (A) Regional SUVRs were obtained from the W. Schiffer rat brain VOI analysis using the PMOD software package (see Methods). (B) No abnormal glucose metabolism of the right anterodorsal hippocampus (arrow) and left entorhinal cortex (arrow head) is seen in a control group. (C, E, F) Decreased glucose metabolism of the left entorhinal cortex but not the right anterodorsal hippocampus is seen in a rat with bilateral CCA ligation after 1 month (CCH group). (D, F) 3 months after bilateral common CCA (CCH group), decreased glucose metabolism is only seen in the right anterodorsal hippocampus. F-18 FDG: F-18 fluorodeoxyglucose; PET: positron emission tomography. SUVR: standardized F-18 FDG uptake values ratio; VOI: volume-of interest; CCA: common carotid artery; CCH: chronic cerebral hypoperfusion.

F-18 FDG PET was performed to evaluate cerebral glucose metabolism in the control and CCH groups 1 month and 3 months after ligation. Regional SUVRs were determined using VOI analysis (Fig 4A). At 1 month after the ligation, the SUVRs of the left entorhinal cortex, bilateral frontal association cortex, right motor cortex, and left somatosensory cortex were significantly lower in the CCH group than those in the control group (*p* = 0.036, *p* = 0.023, *p* = 0.005, and *p* = 0.043, respectively) (Fig 4B–4C and 4E, Table 1). However, there were no significant differences in the SUVR in other regions of the brain at 1 month. At 3 months after the ligation, only the SUVR of the right anterodorsal hippocampus was significantly lower in the CCH group compared with that of the control group (*p* = 0.014) (Fig 4D and 4F, Table 1). There were no significant differences in any other regions between the control and the CCH groups at 3 months after the ligation.

**Table 1:** Comparison of regional cerebral glucose metabolism at 1 and 3 months after surgery.

Regions	Side	1 Month		*p* value	3 Months		*p* value
		Control	CCH		Control	CCH	
Accumbens	left	1.02 (0.09)	0.99 (0.08)	0.589	1.03 (0.11)	0.99 (0.08)	0.312
	right	1.03 (0.08)	0.98 (0.09)	0.206	1.02(0.08)	1.02(0.10)	0.939
Amygdala	left	0.89 (0.05)	0.84 (0.06)	0.070	0.87(0.07)	0.83(0.07)	0.128
	right	0.88 (0.06)	0.87 (0.08)	0.945	0.86(0.06)	0.83(0.05)	0.248
Auditory cortex	left	0.89 (0.09)	0.82 (0.08)	0.084	0.90(0.07)	0.89(0.08)	0.616
	right	0.86 (0.08)	0.79 (0.11)	0.111	0.89(0.05)	0.86(0.07)	0.207
Cingulate cortex	left	1.07 (0.15)	0.95 (0.09)	0.050	1.03(0.11)	1.00(0.12)	0.465
	right	1.05 (0.15)	0.94 (0.10)	0.081	1.04(0.10)	1.01(0.15)	0.503
Entorhinal cortex	left	0.89 (0.06)	0.83 (0.07)	0.036*	0.88(0.06)	0.87(0.08)	0.742
	right	0.89 (0.08)	0.83 (0.07)	0.081	0.87(0.08)	0.88(0.08)	0.752
Frontal association cortex	left	0.95 (0.09)	0.84 (0.09)	0.023*	0.88(0.11)	0.87(0.09)	0.852
	right	0.91 (0.08)	0.78 (0.09)	0.005*	0.87(0.11)	0.86(0.08)	0.655
Insular cortex	left	0.97 (0.10)	0.89 (0.08)	0.059	0.96(0.06)	0.96(0.06)	0.990
	right	0.95 (0.09)	0.89 (0.08)	0.119	0.95(0.08)	0.97(0.09)	0.600
Medial prefrontal cortex	left	1.14 (0.16)	1.04 (0.09)	0.102	1.15(0.14)	1.11(0.17)	0.513
	right	1.10 (0.14)	1.02 (0.10)	0.181	1.14(0.13)	1.13(0.19)	0.970
Motor cortex	left	0.94 (0.10)	0.86 (0.07)	0.088	0.93(0.09)	0.93(0.08)	0.969
	right	0.94 (0.08)	0.85 (0.10)	0.047	0.91(0.07)	0.91(0.09)	0.946
Orbitofrontal cortex	left	1.04 (0.10)	0.96 (0.09)	0.083	1.03(0.09)	1.00(0.09)	0.328
	right	1.01 (0.10)	0.94 (0.08)	0.099	1.02(0.08)	0.98(0.09)	0.246
Para cortex	left	0.85 (0.08)	0.80 (0.04)	0.108	0.88(0.08)	0.88(0.09)	0.944
	right	0.85 (0.08)	0.78 (0.12)	0.151	0.84(0.07)	0.82(0.08)	0.449
Retrosplenial cortex	left	0.93 (0.09)	0.89 (0.08)	0.327	0.94(0.07)	0.92(0.12)	0.674
	right	0.93 (0.08)	0.87 (0.06)	0.118	0.94(0.08)	0.90(0.09)	0.293
Somatosensory cortex	left	0.92 (0.08)	0.85 (0.06)	0.043*	0.92(0.06)	0.93(0.07)	0.535
	right	0.91 (0.06)	0.84 (0.11)	0.095	0.89(0.05)	0.90(0.07)	0.717
Visual cortex	left	0.87 (0.08)	0.81 (0.06)	0.152	0.89(0.09)	0.89(0.09)	0.895
	right	0.86 (0.08)	0.79 (0.10)	0.139	0.85(0.06)	0.83(0.08)	0.641
Anterodorsal hippocampus	left	1.03 (0.07)	0.98 (0.07)	0.160	1.02(0.06)	1.02(0.08)	0.949
	right	1.03 (0.07)	0.99 (0.09)	0.233	1.02(0.07)	0.96(0.09)	0.039*
Posterior hippocampus	left	0.96 (0.06)	0.96 (0.05)	0.831	0.93(0.05)	0.92(0.07)	0.650
	right	0.94 (0.06)	0.96 (0.11)	0.632	0.95(0.06)	0.95(0.09)	0.980

Asterisks indicate statistical significance

*p < 0.05; CCH: chronic cerebral hypoperfusion. All values are presented as mean (standard deviation).

### Development of AD pathology

**Fig 5:**
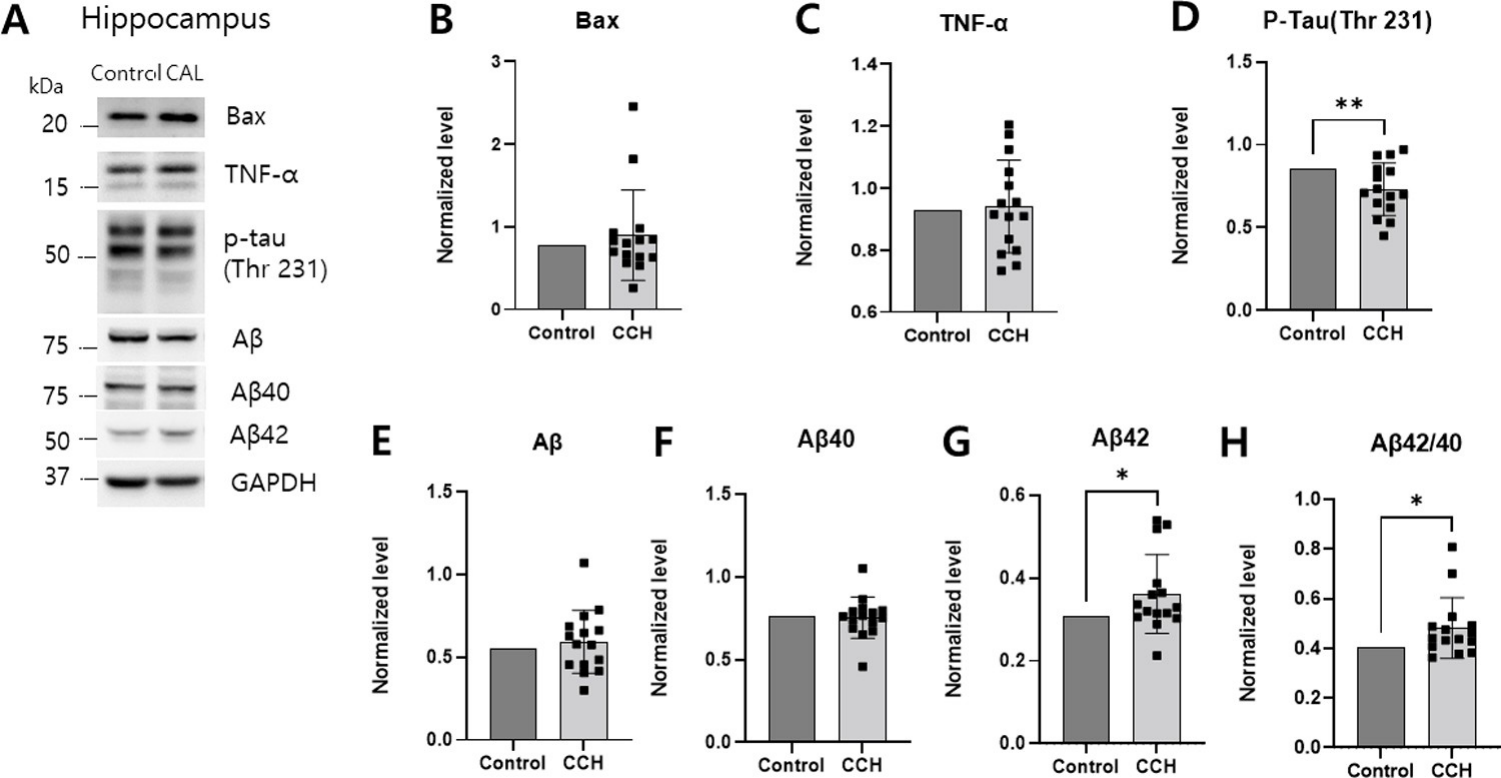
Comparison of Alzheimer’s disease pathology in the hippocampus between the CCH and control groups. (A) The expression levels of Bax, TNF-α, hyperphosphorylated tau (p-tau), amyloid β (Aβ), amyloid β40 (Aβ40), and amyloid β42 (Aβ42) were evaluated in the hippocampus at 3 months after bilateral CCH using western blot analysis. (B-H) The relative ratio of Bax, TNF-α, p-tau, Aβ, Aβ40, and Aβ42 were plotted based on the quantification of band intensity using scion image software. Values are expressed as the mean ± SD. Asterisks indicate statistical significance: *p < 0.05; **p < 0.05. CCH: chronic cerebral hypoperfusion.

Western blot analysis was conducted 3 months after the ligation surgery to determine the protein expression levels of Bax, TNF-α, p-tau, Aβ, Aβ40, Aβ42, and GAPDH in the hippocampus. The expression of Aβ42 was significantly increased in the CCH group compared with that in the control group (*p* = 0.048) (Fig 5). Moreover, the Aβ42/40 ratio in the hippocampus of the CCH group was significantly elevated in comparison to that of the control group (*p* = 0.028). The p-tau levels of the hippocampus in the CCH group were significantly lower as compared with those of the control group (*p* = 0.014). There were no significant differences in the expression levels of Bax, TNF-α, Aβ, and Aβ40 in the hippocampus between the two groups.

### Spatial working memory

**Fig 6:**
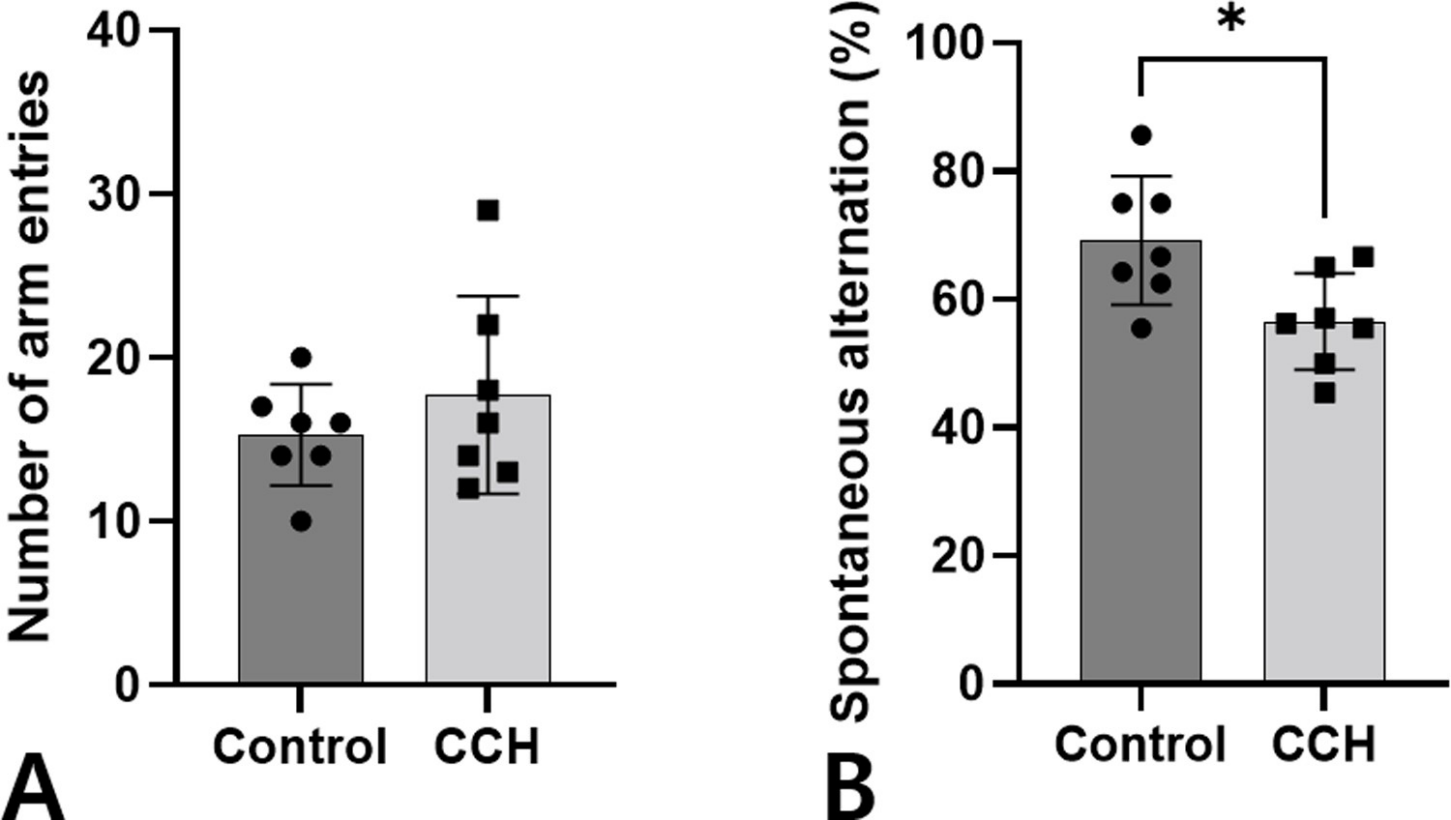
Comparison of spatial working memory assessed by the Y-maze test between the CCH and control groups. The number of arm entries (A) and alternation behaviors (B) measured after 3 months in the bilateral common carotid artery ligation (CCH group) and control group are shown. Asterisks indicate statistical significance: *p < 0.05. CCH: chronic cerebral hypoperfusion.

The Y-maze test was performed 3 months after surgery to assess spatial working memory. There was no difference in the number of arm entries between the control and the CCH groups. In contrast, there was significantly lower spontaneous alternation in the CCH group compared with the control group at 3 (69.25 ± 10.01% vs. 56.58 ± 7.54%, *p* = 0.020; Fig 6).

## Discussion

The present study showed that CCH decreases the neuronal activity of the hippocampus, causing cognitive decline in a rat model. The results also showed that CCH induces cerebral Aβ accumulation. These results suggest that CCH causes selective hippocampal degeneration, which induces AD pathology. The present study helps to understand the mechanism by which cerebrovascular disease contributes to AD development.

CCH by permanent bilateral CCA ligation offers several advantages compared to other approaches. It can induce CCH in a more clinically relevant manner, without ischemic lesions in the brain [24, 25]. The present study induced CCH by CCA ligation in the rat brain using real time CBF monitoring, and there was no ischemic lesion in the brain. The CBF monitoring revealed a sharp decrease in the early stages after surgery, resulting in a 68% and 29% decrease in CBF at 3 and 14 days after the surgery, respectively. The CBF gradually recovered from the 14th day after surgery and reached a normal level at 1 month. In agreement with our results, a previous study with rats reported that bilateral occlusion of the CCA led to a dramatic initial drop in CBF, which subsequently returned to 30–45% CBF in the cortex and a 20% reduction in the hippocampus 1 week after surgery [26]. The gradual recovery of the CBF could be caused by supply from the collateral blood vessels, such as through the posterior communicating artery [20]. In addition, compensation mechanisms for the recovery of CBF may involve biomedical regulation of CBF, recruitment of non-perfused capillaries, and angiogenesis [27]. Unexpectedly, in the present study, the CBF in the CCH group, relative to the control group, increased significantly 3 months after bilateral CCA ligation. This may have been observed because the recruitment of capillaries and angiogenesis can be more pronounced in the cerebral cortex, where the capillary is more developed than in the deep structure, in the CCH rat model [28], and the LDF measured the CBF for a specific region in the cerebral cortex rather than the entire brain [29]. This limitation of LDF is associated with the lack of knowledge of the depth of CBF measurement [29]. In support of this finding, a previous study showed that the percentage and areas of capillaries were increased 6 weeks after bilateral CCA occlusion in the CCH rat model relative to the control group [27]. This was consistent with the findings of another study showing an increase in the capillary density of the cerebral cortex in the chronic cerebral hypoxia mouse model [28]. Another possible reason is that LDF could not accurately evaluate CBF at each time point after bilateral CCA ligation. The LDF may not be suitable for the longitudinal measurement of CBF due to the limitations of LDF, such as the effect of the optical properties of the tissues on the perfusion signal, motion artifact noise, lack of quantitative units for perfusion, and the biological zero signal [29]. To overcome this limitation, we attempted to place the LDF probe in the same position on the skull at each time point.

Several studies of patients with late stage of AD have reported decreased cerebral glucose metabolism in the temporal and parietal cortices, posterior cingulate, and precuneus [30]. Moreover, in the early stage of AD, decreased cerebral glucose metabolism has been observed in the hippocampus, and hippocampal dysfunction has been shown to be associated with that of the parietal and temporal cortices, suggesting that hippocampal degeneration could be an important trigger for the onset of AD [31]. A recent study using high-resolution F-18 FDG PET/MRI reported decreased glucose metabolism in the bilateral hippocampus in patients with early-stage AD [32]. Previous studies using animal models have shown that hippocampal degeneration, observed during the early stage of AD can be caused by CCH [33]. A previous F-18 FDG PET study of a CCH rat model revealed that CCH decreased the glucose metabolism in the hippocampus 3 months after bilateral CCA ligation, inducing AD pathology [18]. Following the previous study, the present study revealed that glucose metabolism in the anterodorsal hippocampus was decreased by CCH 3 months after the ligation. In addition to the previous study, the present study also showed that the decreased glucose metabolism in the cortex 1 month after the ligation improved after 3 months with the recovery of cerebral blood flow; the decrease in glucose metabolism in the hippocampus was observed 3 months after the ligation. These findings support the ischemic hypothesis for AD development related to CCH, showing that the hippocampus is more vulnerable to ischemia than the cortex [34]. It is known that the hippocampus, which is involved in memory formation, is susceptible to ischemia [35]. Another previous F-18 FDG PET study in a CCH mouse model revealed that glucose metabolism in the hippocampus, as well as in the cortex, decreased for up to 6 months after applying microcoils to bilateral CCA, but selective vulnerability to ischemia was not detected in the hippocampus [33]. This may be because the compensation mechanisms for the recovery of CBF were not more extensive in mice than in rats, and the sustained decrease in CBF in the cortex affected the decrease in cerebral glucose metabolism [36, 37].

A previous study of ischemic animal models has revealed that CCH can induce AD pathology [38]. A study using specific enzyme-linked immunosorbent assays reported that white matter hyperintensities were significantly associated with plasma Aβ40 and Aβ42 levels in an AD and mild cognitive impairment population [39]. In agreement with previous studies, the present study showed that the expression levels of Aβ42 and the Aβ42/40 ratio were increased in the hippocampus 3 months after bilateral CCA ligation. Thus, chronic ischemia could contribute to the development of AD through alteration of Aβ metabolism was postulated. In mutant APP transgenic mice, it has been shown that long-term hypoxia contributes to increased Aβ deposition and neuritic plaque formation, potentiating memory deficit by increasing the transcription and expression of the β-site APP cleaving enzyme 1 (BACE1) gene, which is primarily mediated by the binding of hypoxia-inducible factor-1α to the BACE1 promoter [40, 41]. Furthermore, following energy deficiency mediated by pharmacological drugs (e.g. insulin, 2-deoxyglucose, 3-nitropropionic acid, or kainic acid), BACE1 activation and subsent Aβ40 overproduction have been seen in Tg2576 animals [42]. These findings collectively suggest that a lack of energy/oxygen promotes AD pathogenesis by increasing BACE1 expression and Aβ overproduction. Chronic cerebral hypoperfusion has been known to induce the hyperphosphorylation of tau [43, 44]. However, in the present study, the expression of p-tau decreased 3 months after surgery. This discordant result is attributed to the differences in the level of p-tau associated with the clinical features of AD, severity, and the location of the affected brain [45]. Furthermore, the level of p-tau can be measured differently depending on the specific site where phosphorylation occurs, such as at Th205, Th212, Th23, Ser262, Ser396, and Ser404 [43], but only tau phosphorylation at Th231 was measured in the present study. One study involving a mouse model of CCH showed that the level of p-tau increased at Th212 and Ser262 of the cortex, but was not significantly changed at Th231, S262, S396, and S404 [43]. Another study involving an oligemic mouse model of cerebral hypoperfusion revealed a significant decrease in the level of p-tau at Ser199, 202, and Th181, but no significant changes at Th214 [46]. These reductions in the level of p-tau also could be due to the overall reduction in the level of tau rather than specific changes in tau phosphorylation and dephosphorylation [46]. Further longitudinal experiments are needed for the measurement of site-specific tau phosphorylation after CCH.

The Y-maze test can be used to specifically assess short-term memory in rats. Spontaneous alternation, which is a measure of spatial working memory, is especially impaired by AD [47, 48]. The present study revealed that CCH with decreased metabolism in the hippocampus aggravates memory impairment on the Y-maze test. A Rotterdam Study of 1,730 participants suggested that CCH precedes and possibly contributes to the onset of clinical dementia [49]. In an animal study of APPswe/PS1 mice, CCH induced by single vessel occlusion has been shown to exacerbate memory deficits [50, 51]. Another study using the J20/APP AD mouse model showed that CCH induced by bilateral carotid artery stenosis exacerbated learning impairment [13]. The memory impairment induced by CCH is presumed to be caused by hippocampal degeneration. In the early stages of AD, the hippocampus is one of the cerebral structures that undergoes neurodegenerative changes [52, 53]. Hippocampal formation is involved in the learning process and is crucial in in processing and remembering spatial and contextual information [54]. In patients with AD, progressive hippocampal dysfunction has been reported to cause memory impairment [55], which was consistent with our results.

The current study has some limitations. First, the mechanism by which selective hippocampal degeneration causes AD pathology has not been identified. Further, it is still unclear whether CCH generates AD pathology directly or in combination with other causes. Second, our animal model did not have persistent CCH, because the CBF recovered to normal values 1 month after the bilateral CCA ligation surgery. Persistent CCH would likely reveal the effect on AD development more clearly, and would be more consistent with the clinical situation. Animal models of diabetes with a high-fat diet or type-I interferon injection have been shown cause small vessel disease or multivessel atherosclerosis resulting in CCH [56]. Third, this study did not consider the effects of age and gender as only young male Wistar rats were used to evaluate the effects of CCH on AD development. Old age is the strongest risk factor for AD, and the mechanisms for AD development can differ with sex; therefore, evaluating the difference in the effects of chronic cerebral ischemia according to sex in older rats may help elucidate the mechanism of AD development more clearly [57]. Fourth, cognitive function was evaluated by only one behavior test without control tests measuring anxiety-like behavior or locomotor activity. Thus, further studies involving physiological animal models with a complete battery of behavior tests suitable for the clinical situation are needed to establish the effect of CCH on AD development. Finally, the mechanism by which CCH induce AD development, as well as other complicated factors, and how to prevent or slow AD progression by inhibiting this process should be focused on future research.

## Conclusions

Our findings suggest that CCH induces the AD pathology with selectively degeneration of the hippocampus in rats. CCH may play a significant role in the development of AD and the selective neurodegeneration of the hippocampus may be a trigger point for the development of AD pathology. Further studies are needed to elucidate the mechanisms by which CCH aggravates AD pathology.

## Supporting information

S1 File (XLSX)

S2 File (PDF)
