# Supplementary figures and images for: Selective neurodegeneration of the hippocampus caused by chronic cerebral hypoperfusion: F-18 FDG PET study in rats
Source: PLoS One. 2022 Feb 10;17(2):e0262224. doi: 10.1371/journal.pone.0262224 (PMC8830734; doi:10.1371/journal.pone.0262224)

Bax

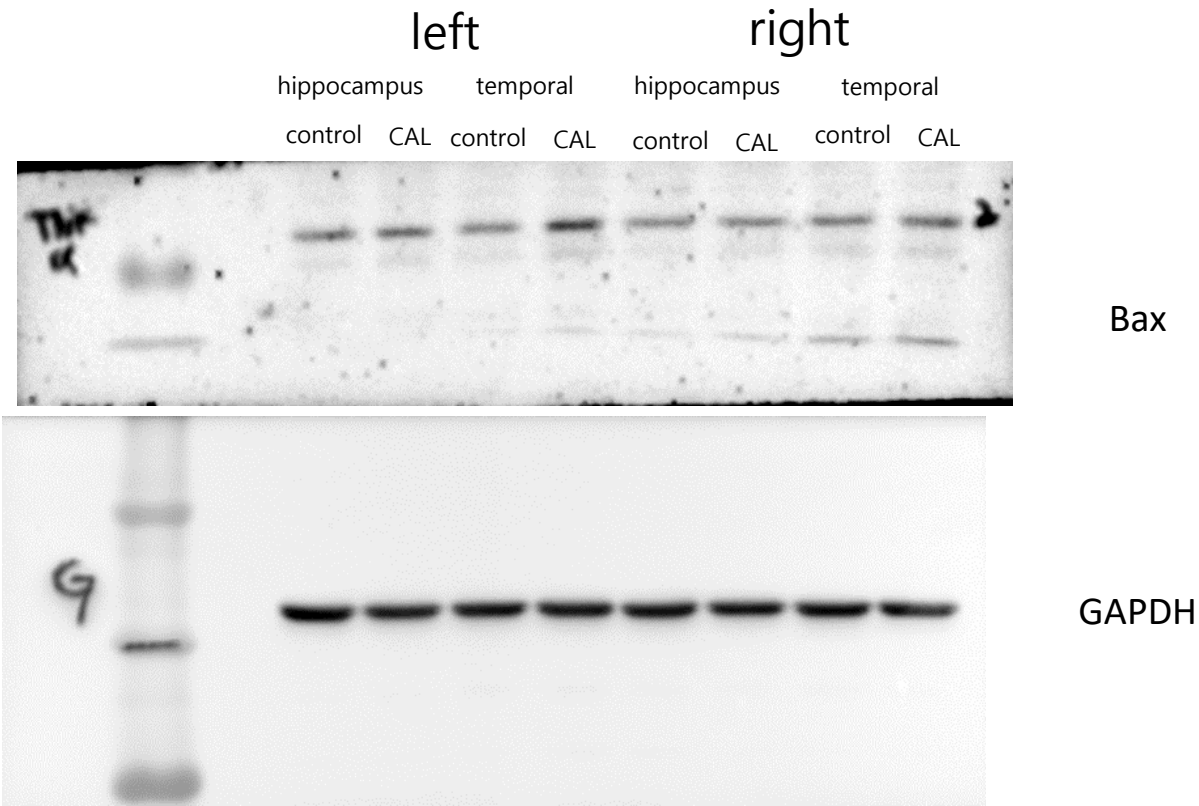

TNF- $\alpha$

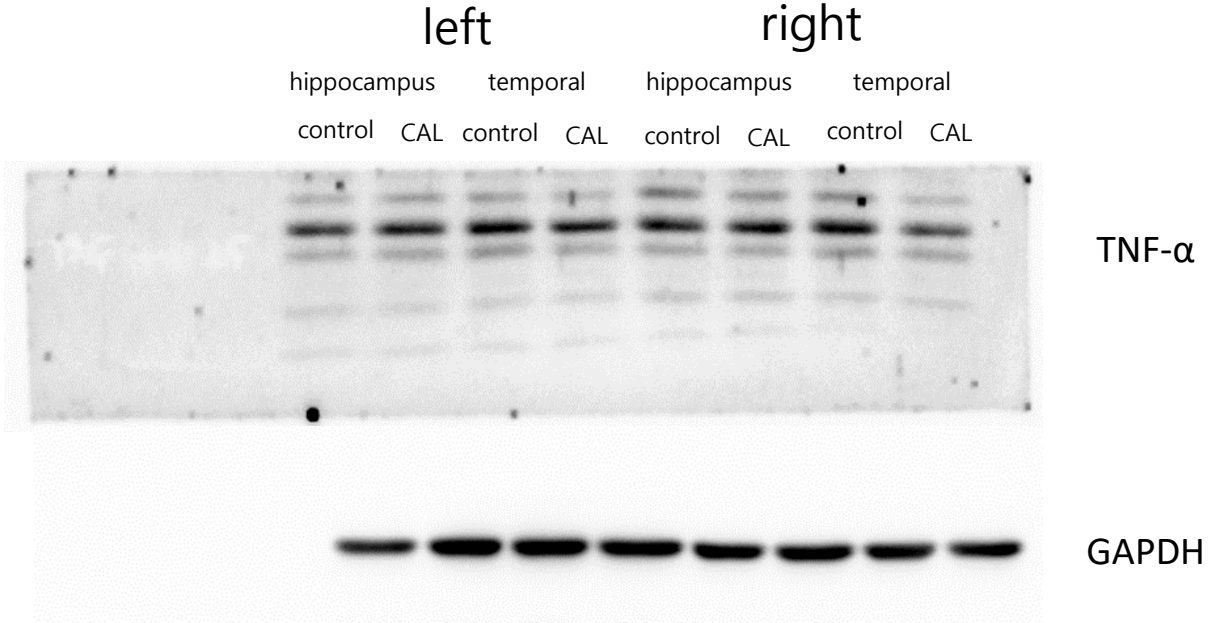

Tau

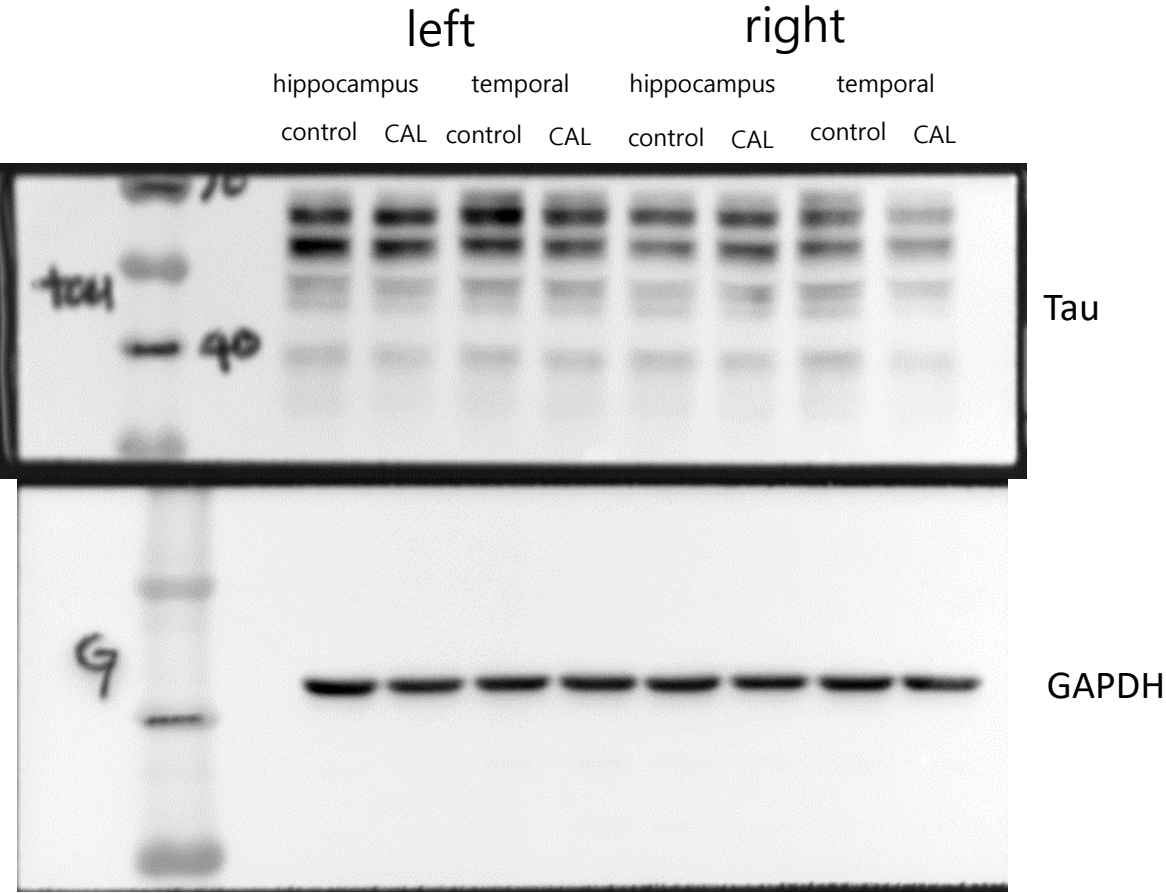

Amyloid  $\beta$

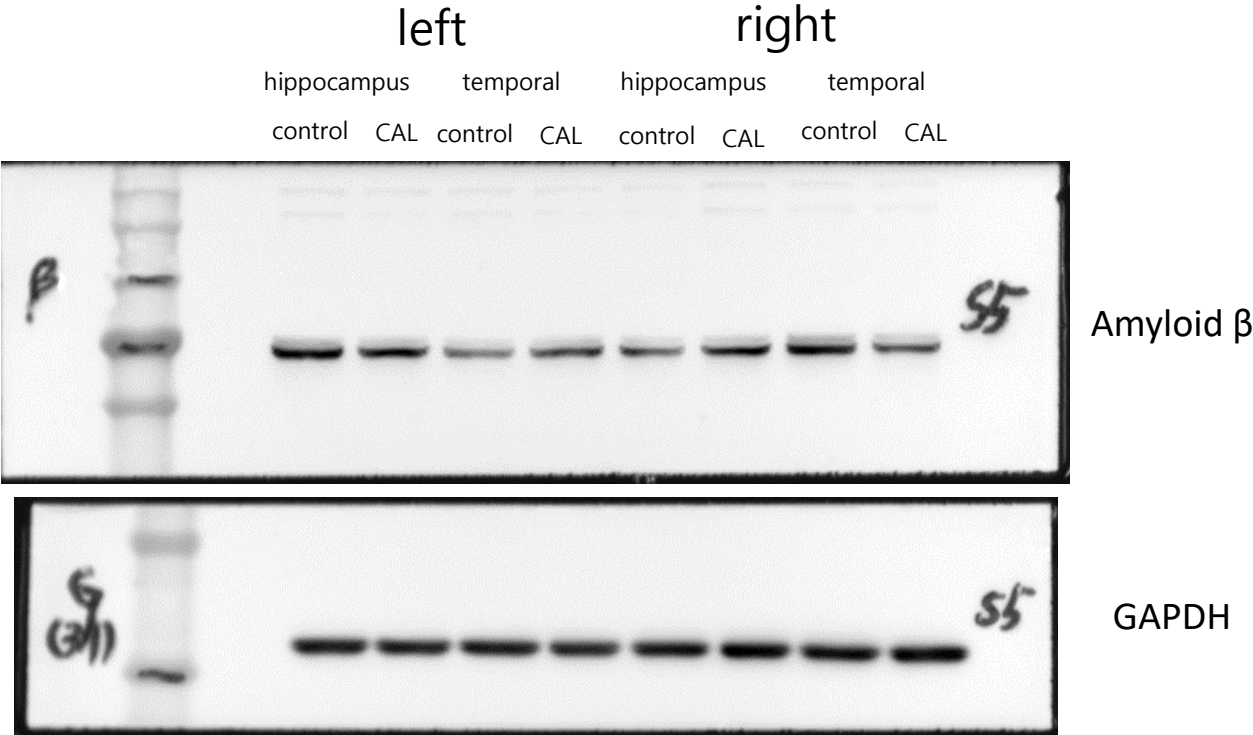

Amyloid  $\beta_{1-40}$

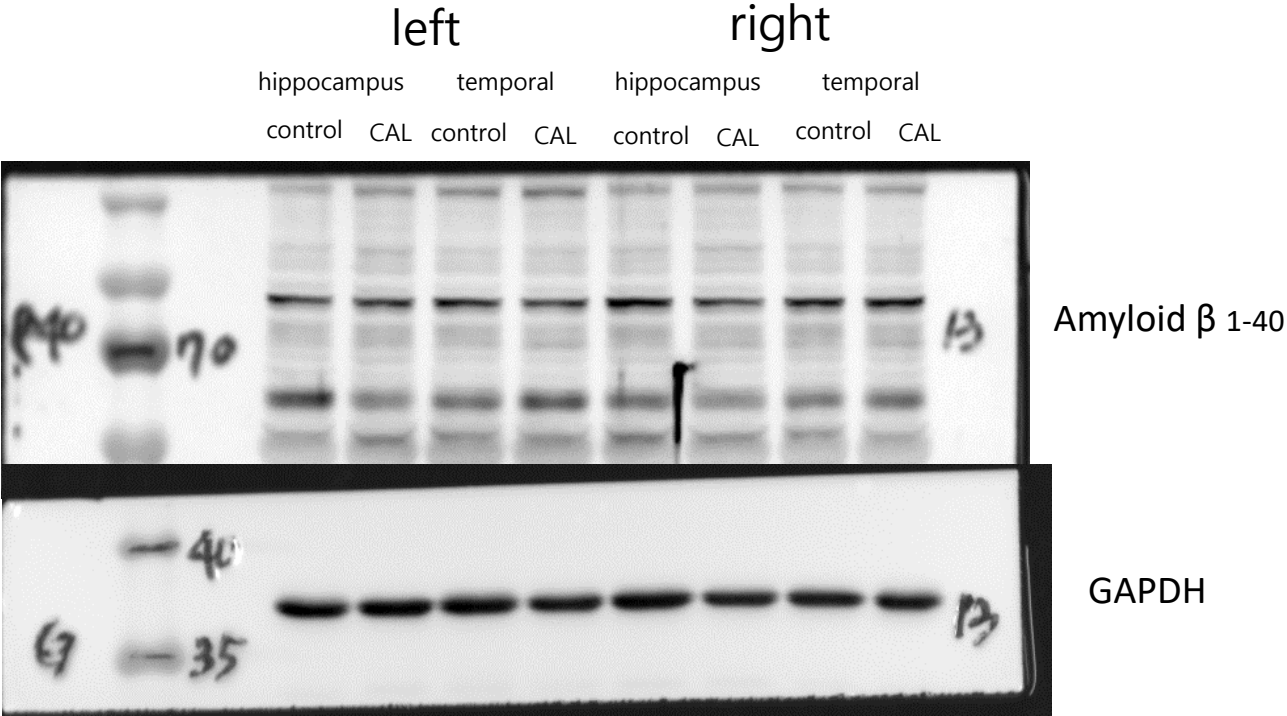

Amyloid  $\beta_{1-42}$

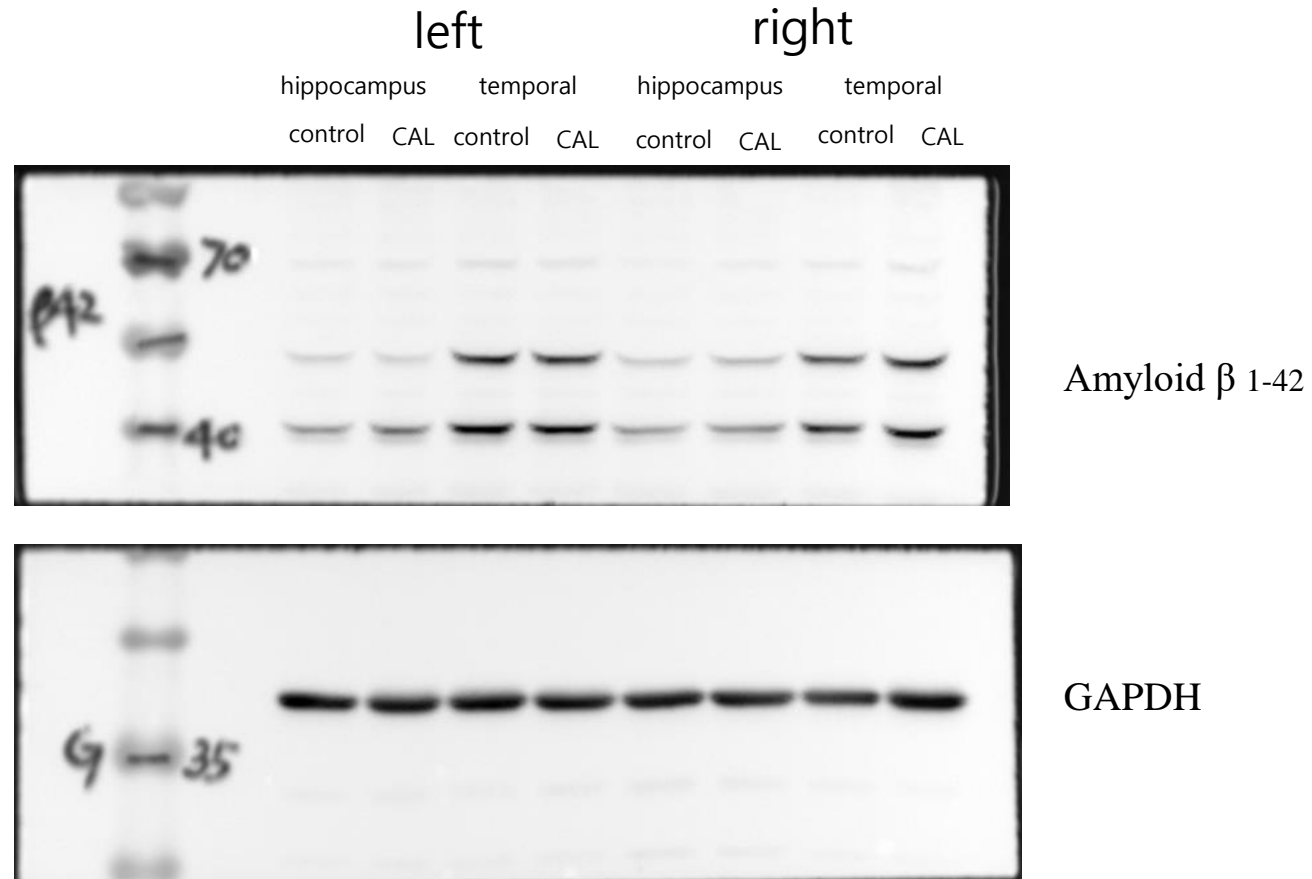

Supplement: S2 File — (PDF) [file pone.0262224.s002.pdf]
