# Supplementary material for: Selective neurodegeneration of the hippocampus caused by chronic cerebral hypoperfusion: F-18 FDG PET study in rats
Source: PLoS One. 2022 Feb 10;17(2):e0262224. doi: 10.1371/journal.pone.0262224 (PMC8830734; doi:10.1371/journal.pone.0262224)
Supplement: Attachment — Submitted filename: Response to Editor1.docx [file pone.0262224.s003.docx]

Response to reviewers

The Editor

*Plos One*

Dear Editor:

Thank you for your careful consideration of our manuscript. We appreciate all your valuable comments. As per your recommendation, we additionally uploaded unedited images of the Western blots and supplemental PDF files containing uncropped, high resolution WB scans. We hope that the revised manuscript will be considered suitable for publication.

Sincerely

Hae Won Kim, MD, PhD

Department of Nuclear Medicine, Keimyung University Dongsan Medical Center

56 Dalseong-ro, Jung-gu, Daegu 41931, Republic of Korea

Tel.: 82-53-250-7068, Fax: 82-53-250-8695, E-mail: [hwkim.nm@gmail.com](mailto:hwkim.nm@gmail.com)
