# Supplementary material for: Selective neurodegeneration of the hippocampus caused by chronic cerebral hypoperfusion: F-18 FDG PET study in rats
Source: PLoS One. 2022 Feb 10;17(2):e0262224. doi: 10.1371/journal.pone.0262224 (PMC8830734; doi:10.1371/journal.pone.0262224)
Supplement: Attachment — Submitted filename: Response_to_Reviewers_2.1.docx [file pone.0262224.s004.docx]

**Response to reviewers**

October 25, 2021

The Editor

*PLOS ONE*

Dear Editor:

I wish to re-submit the manuscript titled “**Selective Neurodegeneration of the Hippocampus Caused by Chronic Cerebral Hypoperfusion: F-18 FDG PET Study in Rats**.”

Thank you for your careful review of our manuscript. We appreciate all your valuable comments and recommendations. The manuscript has benefited from these insightful suggestions. I look forward to working with you and the reviewers to move this manuscript closer to publication in *PLOS ONE.*

All the lapses you highlighted in the manuscript have been corrected, and the corrections are indicated in red text in the revised manuscript. The responses to all comments have been prepared and given below.

1) Reviewer 2 felt that there is an overlap between present study and recent publication in Scientific Reports (J-H Park et al, Sci Rep 9: 14102; 2019).  Please address this concern in detail in your letter to the Editor/responses to Reviewers.  In the Introduction of revised text, please mention outright your previous study and explain how the present work differs from it in terms of addressed questions and newly obtained results.

- We agree with your comment. In a previous study, F-18 FDG PET of a CCH rat model revealed that CCH results in decreased glucose metabolism in the hippocampus, suggesting that CCH can induce AD pathology. However, the previous study did not provide results on cerebral glucose metabolism during the early stage of CCH, and could not be established whether the decreased glucose metabolism in the hippocampus was due to an acute change similar to vascular dementia or a chronic change similar to AD development. In addition, since the previous study assessed cerebral blood flow (CBF) for up to 1 month after CCH, it was difficult to determine how the hippocampus was affected by the improvement in CBF after 3 months. The introduction section has been modified accordingly (page 3, last paragraph).

2) In the Discussion, I have not found any references to your conceptually overlapping work (J-H Park et al, Sci Rep 9: 14102; 2019).  Please address this omission and specify BOTH novel findings and quantitative and qualitative differences from the previous results.  For example, blood flow in CCH animals does not completely recover at 1-month point in the published study but is fully restored in this submission, etc.

- We agree with your comment. The differences between the results of previous studies and the present study are important. In addition to the previous study, the present study showed that the decreased glucose metabolism in the hippocampus was observed 3 months after the ligation, although the decrease in glucose metabolism in the cortex 1 month after the ligation improved after 3 months with the recovery of cerebral blood flow. This finding supports the ischemic hypothesis for AD development related to CCH, showing that the hippocampus is more vulnerable to ischemia than the cortex. The Discussion section has been modified accordingly (page 13, 2^nd^ paragraph).

3) Please address in the text of revised manuscript concern of Reviewer 1 about validity of longitudinal (1 and 3 months) LD CBF measurements.  It seems that capturing LD signal from the same location during long-time frame is technically challenging, to say the least.  Please, specify in the Methods additional technical details of longitudinal LD studies, including thinning of the skull (if any), whether the LD probe was mounted permanently or not, and, if not, how did you cleaned dental resin and assured measurements for the same location.  Reviewer 2 made comments about sensitivity of LD signal to location and limitations of generalized statements based on LD signal.

- We agree with your comment. Laser Doppler flowmetry (LDF) may not be suitable for the longitudinal measurement of CBF due to the limitations of LDF, such as the influence of the optical properties of tissues on the perfusion signal, motion artifact noise, the lack of quantitative units for perfusion, and the biological zero signal. Furthermore, the CBF, which was assessed by LDF, was for specific region in the cerebral cortex rather than the entire brain. Thus, as you commented, we have provided additional technical details on the longitudinal LD studies in the Methods section (page 5, 2^nd^ paragraph) and their limitations in the Discussion section (page 12, 2^nd^ paragraph).

4) I have a question about methodology of detecting amyloid protein processing.  To the best of my understanding, the beta-amyloid antibodies which you use detect monomeric and oligomeric betta amyloid fragments that run at the molecular weights of <10 kD (monomers) and higher molecular weight bands (oligomers). Based on molecular weights of in WB images, I am not sure that you quantify relevant bands. Please address this concern in the letter to the Editor.

- We agree with your valuable comment and checked our antibodies datasheet. We used beta amyloid (abcam, ab216436, MW 87 kDa), beta amyloid 1-40 (abcam, ab17295, MW 87 kDa), and beta amyloid 1-42 (abcam ab201061, MW ~50 kDa for oligomer) antibodies for western blot analysis. We confirmed that the beta amyloid bands had the predicted molecular weights specified in the datasheet. However, in Figure 5, the molecular weight for beta amyloid 1-42 was incorrectly indicated. Thus, the molecular weight for beta amyloid 1-42 was changed to 50 kDa (Figure 5).

We think the reviewers have helped improve the quality of our manuscript by providing appropriate suggestions. We hope that the revised manuscript will be considered suitable for publication.

Thank you for your consideration. I look forward to hearing from you.

Sincerely,

Hae Won Kim, MD, PhD

Department of Nuclear Medicine, Keimyung University Dongsan Medical Center

56 Dalseong-ro, Jung-gu, Daegu 41931, Republic of Korea

Tel.: 82-53-250-7068, Fax: 82-53-250-8695, E-mail: [hwkim.nm@gmail.com](mailto:hwkim.nm@gmail.com)

**To Reviewer #1**

Thank you for your careful comments. The corrections are indicated by red-font text in the revised manuscript.

1) In the structured Abstract, the authors referred to hyperphosphorylated tau (p-tau) in the Methods but not in the Results. The p-tau (Thr 231) was significantly reduced in the CCH group. Please discuss this potentially interesting result in the Abstract and text.
- As you recommended, we have provided the results of p-tau in the Abstract. Possible reasons for the discordant results for the p-tau level have also been provided in the Discussion section (page 14, 1^st^ paragraph).

2) The authors found significant increase of CBF in the CCH group at 3 months. Longitudinal measurement of CBF is thought not to be accurate at 1 month and 3 months after surgery. Please discuss this. The authors stated that the gradual recovery of the CBF could be caused by supply from the collateral blood vessels, such as through the posterior communicating artery. This is a plausible explanation of the result shown in Figure 2. However, do collateral blood vessels induce significant increase of the CBF at 3 months? Please discuss the underlying mechanism of the overshoot of compensatory responses.

- We agree with your comment. The Laser Doppler flowmetry (LDF) may not suitable for the longitudinal measurement of CBF due to the limitations of LDF, such as the influence of the optical properties of tissues on the perfusion signal, motion artifact noise, lack of quantitative units for perfusion, and the biological zero signal. In addition to these limitations of LDF, the possible underlying mechanisms of the overshoot of compensatory responses are described in the Discussion section (page 12, 2^nd^ paragraph).

3) An important reference is not included. FDG-PET study in a mouse model of CCH was previously reported in PMID: 20448204. This should be included in the references and should be thoroughly discussed regarding similarities and differences in the findings between mice and rats.

- We agree with your insightful comment. A previous study involving the CCH mouse model, which you mentioned, reported that glucose metabolism in the hippocampus, as well as in the cortex, decreased for up to 6 months after bilateral CCA ligation, failing to prove the selective vulnerability to ischemia in the hippocampus. This differs from our results showing that the hippocampus is more vulnerable to ischemia than the cortex. The following may account for the difference in results: the compensation mechanisms for recovery of CBF did not occur more extensively in mice than in rats, and the decreased CBF in the cortex affected the decrease in cerebral glucose metabolism. The Discussion section has been updated accordingly (page 13, 2^nd^ paragraph).

**To Reviewer #2**

Thank you for your careful comments. The corrections are indicated by red-font in the revised manuscript.

1) It is unclear how this study is sufficiently different from the previously published work, except for addition of the 1 month time point of glucose metabolism.

- We agree with your comment. It is important to note the differences between the results of previous studies and the present study. A previous F-18 FDG PET study of a CCH rat model revealed that CCH decreased the glucose metabolism in the hippocampus 3 months after bilateral CCA ligation, inducing AD pathology. Following the previous study, the present study revealed that glucose metabolism in the anterodorsal hippocampus was decreased by CCH 3 months after the ligation. In addition to the previous study, the present study also showed that the decreased glucose metabolism in the cortex 1 month after the ligation improved after 3 months with the recovery of cerebral blood flow; the decrease in glucose metabolism in the hippocampus was observed 3 months after the ligation. These findings support the ischemic hypothesis for AD development related to CCH, showing that the hippocampus is more vulnerable to ischemia than the cortex. The Introduction and Discussion sections have been updated accordingly (page 3, last paragraph and page 13, 2^nd^ paragraph).

2) Sex and age are not appropriately considered ask key biological variables. Only young males are used. Dementia is a disease of aging, so use of young animals should be discussed as a limitation. Further, use of only one sex should be discussed as a major limitation.

- Old age is the strongest risk factor for AD, and the mechanisms for AD development can differ with sex; therefore, evaluating the differences in the effects of chronic cerebral ischemia according to sex in older rats may help elucidate the mechanism of AD development more clearly. This limitation has been added to the Discussion section (page 15, last paragraph).

3) Behavior test results are inaccurately described. Y maze is described as an assessment of learning and recognition memory. However, as tested, the Y maze protocol used assesses working memory. The test should be revised.

- As you mentioned, the Y-maze test can be used to assess short-term working memory in rats. We have revised the manuscript to highlight the characteristics of the Y-maze test in the Methods and Discussion sections (page 14, last paragraph).

4) Use of only 1 behavior tests with no control tests that assessed anxiety-like behavior or locomotor activity is a limitation.

- We agree that is a limitation of the present study. We have added it to limitation in the Discussion section (page 15, last paragraph).

5) Use of laser Doppler probe for blood flow measurement should be discussed as a limitation. This method allows assessment of only one very small regional of interest and may not reflect a lack of cerebral hypoperfsion in the entire brain at this time point.

- We agree with your comment. The CBF, which was measured by Laser Doppler flowmetry, was for a specific region in the cerebral cortex rather than the entire brain. This is related to the limitation of Laser Doppler flowmetry, with lack of the knowledge of the depth of CBF measurement. We have highlighted this limitation in the Discussion section (page 13, 1^st^ paragraph).

6) Graphs could be improved. Some are pixelated and none include the individual data points.

- Figures 4, 5, and 6 have been modified to include individual data points.

7) Please mention whether you are assessing soluble or insoluble amyloid beta.

- Western blots were used to assess the expressions of soluble Aβ, Aβ40, and Aβ42. We have described this in the Methods section (page 6, last paragraph).

8) Please add lot numbers for primary antibodies.

- We agree with your comment. It is important to record the lot number, because the same primary antibody may yield different results depending on the factory, date of manufacture, and raw material. Unfortunately, we did not record the lot number. We are sorry for not providing the lot number.

9) Please reference table 1 (in addition to 4A-B) when describing differences in cerebral metabolism.

- As you suggested, we have added Table 1 and Figure 4A-B to provide further details on the differences in cerebral metabolism in the Results section (page 9, 1^st^ paragraph).
