# Supplementary material for: Selective neurodegeneration of the hippocampus caused by chronic cerebral hypoperfusion: F-18 FDG PET study in rats
Source: PLoS One. 2022 Feb 10;17(2):e0262224. doi: 10.1371/journal.pone.0262224 (PMC8830734; doi:10.1371/journal.pone.0262224)
Supplement: Attachment — Submitted filename: Response_to_Reviewers_3.0.docx [file pone.0262224.s005.docx]

**Response to reviewers**

December 13, 2021

The Editor

*PLOS ONE*

Dear Editor:

I wish to re-submit the manuscript titled “**Selective Neurodegeneration of the Hippocampus Caused by Chronic Cerebral Hypoperfusion: F-18 FDG PET Study in Rats**.”

Thank you for your careful review of our manuscript. We appreciate all your valuable comments and recommendations. The manuscript has benefited from these insightful suggestions. I look forward to working with you and the reviewers to move this manuscript closer to publication in *PLOS ONE.*

All the lapses you highlighted in the manuscript have been corrected, and the corrections are indicated in red text in the revised manuscript. The responses to all comments have been prepared and are given below.

1) Reviewer 1 has a valid point about the effects of bilateral CCA ligation in MICE, which are misquoted in your manuscript on p. 13.  Unlike rats, many strains of mice have low patency of the posterior communicators.  The original paper, which tested the effects of bilateral CCA ligations in mice (K Kitawa et al, JCBFM, 1998), identified robust forebrain/hippocampal damage after only 15 min of bilateral CCA ligation, particularly in C56BL/6.  Indeed, I think that the manuscript by Kitawa et al. showing incomplete and variable patency of posterior communicator arteries in mice strains has a place in your discussion.  It highlights the difference in perfusion patterns between species with pathological implications.

**Response:** We agree with your insightful comment. Nishio et al. reported the effects of chronic cerebral hypoperfusion in mice with bilateral common carotid artery stenosis using microcoils. That sentence has been modified as Reviewer 1 commented (page 3, 2nd paragraph).

2) Reviewer 2 requested additional clarity on statistical analysis.  Specifically, you need to clarify which test was used for analyzing differences in western blot immunoreactivities.  These cannot be analyzed using two populations t-test, but rather one-population t-test where values are compared to the unity.  If this is not the case, please re-analyze the data and make appropriate corrections in the text.

**Response:** We agree with your insightful comment. The expression levels for Bax, TNF-α, p-tau, Aβ, Aβ40, and Aβ42 in the CCH group were normalized to the average of the control group and were compared with those of the control group using one-sample t-test. The results of western blotting using one-sample t-test did not differ from the previous results, so the *p* value was slightly modified. The statistical analysis method used for the western blotting results was modified in the statistical analysis section (page 7, last paragraph), and Figure 5 was also modified accordingly.

We think the reviewers have helped improve the quality of our manuscript by providing appropriate suggestions. We hope that the revised manuscript will be considered suitable for publication.

Thank you for your consideration. I look forward to hearing from you.

Sincerely,

Hae Won Kim, MD, PhD

Department of Nuclear Medicine, Keimyung University Dongsan Medical Center

56 Dalseong-ro, Jung-gu, Daegu 41931, Republic of Korea

Tel.: 82-53-250-7068, Fax: 82-53-250-8695, E-mail: [hwkim.nm@gmail.com](mailto:hwkim.nm@gmail.com)

**To Reviewer #1**

Thank you for your careful comments. The corrections are indicated by red-font text in the revised manuscript.

1) The authors stated that ‘Another previous F-18 FDG PET study involving a CCH mouse model revealed that glucose metabolism in the hippocampus, as well as in the cortex, decreased for up to 6 months after bilateral CCA ligation, failing to prove the selective vulnerability to the ischemia in the hippocampus [33].’ This sentence is not accurate because a CCH mouse model is generated by bilateral CCA stenosis with microcoils instead of ligation. Therefore, please correct it accordingly. In addition, it would be better to replace ‘failing to prove’ by ‘but did not show’.

**Response:** We agree with your insightful comment. Nishio et al. reported the effects of chronic cerebral hypoperfusion in mice with bilateral common carotid artery stenosis using microcoils. That sentence has been modified as you commented (page 13, 2nd paragraph).

**To Reviewer #2**

Thank you for your careful comments. The corrections are indicated by red-font in the revised manuscript.

1) In adding individual data points to the graph, I now see additional issues with rigor. The analysis in Figure 5 is inappropriate. There are no data points for the control group because it was all normalized as 1. There should be normalization to the average of the control group so that there is still an ability to plot and see the variation in individual data points within the controls. Please correct this.

**Response:** We agree with your insightful comment. The expression levels for Bax, TNF-α, p-tau (Thr231), Aβ, Aβ40 and Aβ42 in the CCH group were normalized to the average of the control group, and were compared with those of the control group using one-sample t-test. The results of western blotting using one-sample t-test did not differ from the previous results, so the *p* value was slightly modified. The statistical analysis method used for the western blotting results was modified in the statistical analysis section (page 7, last paragraph), and Figure 5 was also modified accordingly.
