# Supplementary material for: Selective neurodegeneration of the hippocampus caused by chronic cerebral hypoperfusion: F-18 FDG PET study in rats
Source: PLoS One. 2022 Feb 10;17(2):e0262224. doi: 10.1371/journal.pone.0262224 (PMC8830734; doi:10.1371/journal.pone.0262224)
Supplement: Peer review history

# Editor report: Decision Letter 0

**DOI:** 10.1371/journal.pone.0262224.r001

**Authors:** Alexander A. Mongin

27 Aug 2021

PONE-D-21-25820

Selective Neurodegeneration of the Hippocampus Caused by Chronic Cerebral Hypoperfusion: F-18 FDG PET Study in Rats

PLOS ONE

Dear Dr. Kim,

Thank you for submitting your manuscript to PLOS ONE. After preliminary editorial evaluation, I found that this study has merit but does not fully meet PLOS ONE’s publication criteria as it currently stands.

Specifically, per PLOS ONE policies submitted manuscripts should include quantitative primary data in a format allowing for their re-evaluation (such as in MS Excel spreadsheet). Your can provide these data in a supplemental file.  Reviewers should also be able to inspect the primary, unedited images of your Western blots, which have been used for preparing Fig. 5.  These can be included as a supplemental PDF file containing uncropped, high resolution WB scans.

Therefore, I invite you to submit a revised version of the manuscript that addresses the points raised during the preliminary review process.  As soon as I have the requested additional information, your manuscript will be forwarded to experts in the field for peer review. 

Please submit your revised manuscript as soon as possible but not late than by Oct 03 2021 11:59PM. If you will need more time than this to complete your revisions, please reply to this message or contact the journal office at plosone@plos.org. When you're ready to submit your revision, log on to https://www.editorialmanager.com/pone/ and select the 'Submissions Needing Revision' folder to locate your manuscript file.

If you would like to make changes to your financial disclosure, please include your updated statement in your cover letter. Guidelines for resubmitting your figure files are available below the reviewer comments at the end of this letter.

If applicable, we recommend that you deposit your laboratory protocols in protocols.io to enhance the reproducibility of your results. Protocols.io assigns your protocol its own identifier (DOI) so that it can be cited independently in the future. For instructions see: http://journals.plos.org/plosone/s/submission-guidelines#loc-laboratory-protocols. Additionally, PLOS ONE offers an option for publishing peer-reviewed Lab Protocol articles, which describe protocols hosted on protocols.io. Read more information on sharing protocols at https://plos.org/protocols?utm_medium=editorial-email&utm_source=authorletters&utm_campaign=protocols.

We look forward to receiving your revised manuscript.

Kind regards,

Alexander A. Mongin, Ph.D.

Academic Editor

PLOS ONE

Journal Requirements:

When submitting your revision, we need you to address these additional requirements.

1. Please ensure that your manuscript meets PLOS ONE's style requirements, including those for file naming. The PLOS ONE style templates can be found at 

https://journals.plos.org/plosone/s/file?id=wjVg/PLOSOne_formatting_sample_main_body.pdf and 

https://journals.plos.org/plosone/s/file?id=ba62/PLOSOne_formatting_sample_title_authors_affiliations.pdf

2. We note that you have indicated that data from this study are available upon request. PLOS only allows data to be available upon request if there are legal or ethical restrictions on sharing data publicly. For more information on unacceptable data access restrictions, please see http://journals.plos.org/plosone/s/data-availability#loc-unacceptable-data-access-restrictions. 

In your revised cover letter, please address the following prompts:

a) If there are ethical or legal restrictions on sharing a de-identified data set, please explain them in detail (e.g., data contain potentially sensitive information, data are owned by a third-party organization, etc.) and who has imposed them (e.g., an ethics committee). Please also provide contact information for a data access committee, ethics committee, or other institutional body to which data requests may be sent.

b) If there are no restrictions, please upload the minimal anonymized data set necessary to replicate your study findings as either Supporting Information files or to a stable, public repository and provide us with the relevant URLs, DOIs, or accession numbers. For a list of acceptable repositories, please see http://journals.plos.org/plosone/s/data-availability#loc-recommended-repositories.

We will update your Data Availability statement on your behalf to reflect the information you provide.

3. PLOS ONE now requires that authors provide the original uncropped and unadjusted images underlying all blot or gel results reported in a submission’s figures or Supporting Information files. This policy and the journal’s other requirements for blot/gel reporting and figure preparation are described in detail at https://journals.plos.org/plosone/s/figures#loc-blot-and-gel-reporting-requirements and https://journals.plos.org/plosone/s/figures#loc-preparing-figures-from-image-files. When you submit your revised manuscript, please ensure that your figures adhere fully to these guidelines and provide the original underlying images for all blot or gel data reported in your submission. See the following link for instructions on providing the original image data: https://journals.plos.org/plosone/s/figures#loc-original-images-for-blots-and-gels. 

  

In your cover letter, please note whether your blot/gel image data are in Supporting Information or posted at a public data repository, provide the repository URL if relevant, and provide specific details as to which raw blot/gel images, if any, are not available. Email us at plosone@plos.org if you have any questions.

4. Your ethics statement should only appear in the Methods section of your manuscript. If your ethics statement is written in any section besides the Methods, please delete it from any other section.

[Note: HTML markup is below. Please do not edit.]

Reviewers' comments:

[NOTE: If reviewer comments were submitted as an attachment file, they will be attached to this email and accessible via the submission site. Please log into your account, locate the manuscript record, and check for the action link "View Attachments". If this link does not appear, there are no attachment files.]

While revising your submission, please upload your figure files to the Preflight Analysis and Conversion Engine (PACE) digital diagnostic tool, https://pacev2.apexcovantage.com/. PACE helps ensure that figures meet PLOS requirements. To use PACE, you must first register as a user. Registration is free. Then, login and navigate to the UPLOAD tab, where you will find detailed instructions on how to use the tool. If you encounter any issues or have any questions when using PACE, please email PLOS at figures@plos.org. Please note that Supporting Information files do not need this step.

# Author comment: Author response to Decision Letter 0

**DOI:** 10.1371/journal.pone.0262224.r002

14 Sep 2021

Dear Editor:

Thank you for your careful consideration of our manuscript. We appreciate all your valuable comments. As per your recommendation, we additionally uploaded unedited images of the Western blots and supplemental PDF files containing uncropped, high resolution WB scans. We hope that the revised manuscript will be considered suitable for publication.

Sincerely

Hae Won Kim, MD, PhD

Department of Nuclear Medicine, Keimyung University Dongsan Medical Center 

56 Dalseong-ro, Jung-gu, Daegu 41931, Republic of Korea

Tel.: 82-53-250-7068, Fax: 82-53-250-8695, E-mail: hwkim.nm@gmail.com

Attachment Submitted filename: Response to Editor1.docx Click here for additional data file.

# Peer review: Decision Letter 1

**DOI:** 10.1371/journal.pone.0262224.r003

**Authors:** Alexander A. Mongin

24 Sep 2021

PONE-D-21-25820R1Selective Neurodegeneration of the Hippocampus Caused by Chronic Cerebral Hypoperfusion: F-18 FDG PET Study in RatsPLOS ONE

Dear Dr. Kim,

Thank you for submitting your manuscript to PLOS ONE. Your work has been evaluated by two experts in the field. After careful consideration, we feel that several serious concerns need to be address in order to assure that manuscript meets PLOS ONE’s publication criteria. We invite you to submit a revised version of the manuscript that addresses the points raised during the review process.

 

Specifically, I would like to attract your attention to the following important points:

[1] Reviewer 2 felt that there is an overlap between present study and recent publication in Scientific Reports (J-H Park et al, Sci Rep 9: 14102; 2019).  Please address this concern in detail in your letter to the Editor/responses to Reviewers.  In the Introduction of revised text, please mention outright your previous study and explain how the present work differs from it in terms of addressed questions and newly obtained results.

[2] In the Discussion, I have not found any references to your conceptually overlapping work (J-H Park et al, Sci Rep 9: 14102; 2019).  Please address this omission and specify BOTH novel findings and quantitative and qualitative differences from the previous results.  For example, blood flow in CCH animals does not completely recover at 1-month point in the published study but is fully restored in this submission, etc.

[3] Please address in the text of revised manuscript concern of Reviewer 1 about validity of longitudinal (1 and 3 months) LD CBF measurements.  It seems that capturing LD signal from the same location during long-time frame is technically challenging, to say the least.  Please, specify in the Methods additional technical details of longitudinal LD studies, including thinning of the skull (if any), whether the LD probe was mounted permanently or not, and, if not, how did you cleaned dental resin and assured measurements for the same location.  Reviewer 2 made comments about sensitivity of LD signal to location and limitations of generalized statements based on LD signal. 

[4] I have a question about methodology of detecting amyloid protein processing.  To the best of my understanding, the beta-amyloid antibodies which you use detect monomeric and oligomeric betta amyloid fragments that run at the molecular weights of <10 kD (monomers) and higher molecular weight bands (oligomers).  Based on molecular weights of in WB images, I am not sure that you quantify relevant bands .  Please address this concern in the letter to the Editor.

Please submit your revised manuscript by Nov 08 2021 11:59PM. If you will need more time than this to complete your revisions, please reply to this message or contact the journal office at plosone@plos.org. When you're ready to submit your revision, log on to https://www.editorialmanager.com/pone/ and select the 'Submissions Needing Revision' folder to locate your manuscript file.

Please include the following items when submitting your revised manuscript:

- A rebuttal letter that responds to each point raised by the academic editor and reviewer(s). You should upload this letter as a separate file labeled 'Response to Reviewers'.
- A marked-up copy of your manuscript that highlights changes made to the original version. You should upload this as a separate file labeled 'Revised Manuscript with Track Changes'.
- An unmarked version of your revised paper without tracked changes. You should upload this as a separate file labeled 'Manuscript'.

If you would like to make changes to your financial disclosure, please include your updated statement in your cover letter. Guidelines for resubmitting your figure files are available below the reviewer comments at the end of this letter.

If applicable, we recommend that you deposit your laboratory protocols in protocols.io to enhance the reproducibility of your results. Protocols.io assigns your protocol its own identifier (DOI) so that it can be cited independently in the future. For instructions see: https://journals.plos.org/plosone/s/submission-guidelines#loc-laboratory-protocols. Additionally, PLOS ONE offers an option for publishing peer-reviewed Lab Protocol articles, which describe protocols hosted on protocols.io. Read more information on sharing protocols at https://plos.org/protocols?utm_medium=editorial-email&utm_source=authorletters&utm_campaign=protocols.

We look forward to receiving your revised manuscript.

Kind regards,

Alexander A. Mongin, Ph.D.

Academic Editor

PLOS ONE

[Note: HTML markup is below. Please do not edit.]

Reviewers' comments:

Reviewer's Responses to Questions

**Comments to the Author**

1. If the authors have adequately addressed your comments raised in a previous round of review and you feel that this manuscript is now acceptable for publication, you may indicate that here to bypass the “Comments to the Author” section, enter your conflict of interest statement in the “Confidential to Editor” section, and submit your "Accept" recommendation.

Reviewer #1: (No Response)

Reviewer #2: (No Response)

**********

2. Is the manuscript technically sound, and do the data support the conclusions?

The manuscript must describe a technically sound piece of scientific research with data that supports the conclusions. Experiments must have been conducted rigorously, with appropriate controls, replication, and sample sizes. The conclusions must be drawn appropriately based on the data presented. 

Reviewer #1: Partly

Reviewer #2: Yes

**********

3. Has the statistical analysis been performed appropriately and rigorously? 

Reviewer #1: Yes

Reviewer #2: Yes

**********

4. Have the authors made all data underlying the findings in their manuscript fully available?

The PLOS Data policy requires authors to make all data underlying the findings described in their manuscript fully available without restriction, with rare exception (please refer to the Data Availability Statement in the manuscript PDF file). The data should be provided as part of the manuscript or its supporting information, or deposited to a public repository. For example, in addition to summary statistics, the data points behind means, medians and variance measures should be available. If there are restrictions on publicly sharing data—e.g. participant privacy or use of data from a third party—those must be specified.

Reviewer #1: Yes

Reviewer #2: Yes

**********

5. Is the manuscript presented in an intelligible fashion and written in standard English?

PLOS ONE does not copyedit accepted manuscripts, so the language in submitted articles must be clear, correct, and unambiguous. Any typographical or grammatical errors should be corrected at revision, so please note any specific errors here.

Reviewer #1: Yes

Reviewer #2: Yes

**********

6. Review Comments to the Author

Please use the space provided to explain your answers to the questions above. You may also include additional comments for the author, including concerns about dual publication, research ethics, or publication ethics. (Please upload your review as an attachment if it exceeds 20,000 characters)

Reviewer #1: The authors tried to identify the cerebral regions that are affected by chronic cerebral hypoperfusion (CCH), and to evaluate the development of Alzheimer pathology in a rat model of CCH. The findings are interesting, but there are some methodological and interpretational flaws that should be amended.

In the structured Abstract, the authors referred to hyperphosphorylated tau (p-tau) in the Methods but not in the Results. The p-tau (Thr 231) was significantly reduced in the CCH group. Please discuss this potentially interesting result in the Abstract and text.

The authors found significant increase of CBF in the CCH group at 3 months. Longitudinal measurement of CBF is thought not to be accurate at 1 month and 3 months after surgery. Please discuss this.

The authors stated that the gradual recovery of the CBF could be caused by supply from the collateral blood vessels, such as through the posterior communicating artery. This is a plausible explanation of the result shown in Figure 2. However, do collateral blood vessels induce significant increase of the CBF at 3 months? Please discuss the underlying mechanism of the overshoot of compensatory responses.

An important reference is not included. FDG-PET study in a mouse model of CCH was previously reported in PMID: 20448204. This should be included in the references and should be thoroughly discussed regarding similarities and differences in the findings between mice and rats.

Reviewer #2: The study is well powered and offers an assessment of cerebral glucose metabolism and cerebral blood flow at multiple time points. Unfortunately, novelty is lacking. The blood flow time course has already been published, by this group and others. Further, they have already published the cerebral glucose metabolism and neuropathology data at the 3 month time point. Thus, very little new information is presented.

Comments:

• It is unclear how this study is sufficiently different from the previously published work, except for addition of the 1 month time point of glucose metabolism (https://www.nature.com/articles/s41598-019-50681-4#Sec10).

• Sex and age are not appropriately considered ask key biological variables. Only young males are used. Dementia is a disease of aging, so use of young animals should be discussed as a limitation. Further, use of only one sex shoudl be discussed as a major limitation.

• Behavior test results are inaccurately described. Y maze is described as an assessment of learning and recognition memory. However, as tested, the Y maze protocol used assesses working memory. The test should be revised.

• Use of only 1 behavior tests with no control tests that assessed anxiety-like behavior or locomotor activity is a limitation.

• Use of laser Doppler probe for blood flow measurement should be discussed as a limitation. This method allows assessment of only one very small regional of interest and may not reflect a lack of cerebral hypoperfsion in the entire brain at this time point.

• Graphs could be improved. Some are pixelated and none include the individual data points.

• Please mention whether you are assessing soluble or insoluble amyloid beta.

• Please add lot numbers for primary antibodies.

• Please reference table 1 (in addition to 4A-B) when describing differences in cerebral metabolism.

**********

7. PLOS authors have the option to publish the peer review history of their article (what does this mean?). If published, this will include your full peer review and any attached files.

If you choose “no”, your identity will remain anonymous but your review may still be made public.

**Do you want your identity to be public for this peer review?** For information about this choice, including consent withdrawal, please see our Privacy Policy.

Reviewer #1: No

Reviewer #2: No

[NOTE: If reviewer comments were submitted as an attachment file, they will be attached to this email and accessible via the submission site. Please log into your account, locate the manuscript record, and check for the action link "View Attachments". If this link does not appear, there are no attachment files.]

While revising your submission, please upload your figure files to the Preflight Analysis and Conversion Engine (PACE) digital diagnostic tool, https://pacev2.apexcovantage.com/. PACE helps ensure that figures meet PLOS requirements. To use PACE, you must first register as a user. Registration is free. Then, login and navigate to the UPLOAD tab, where you will find detailed instructions on how to use the tool. If you encounter any issues or have any questions when using PACE, please email PLOS at figures@plos.org. Please note that Supporting Information files do not need this step.

# Author comment: Author response to Decision Letter 1

**DOI:** 10.1371/journal.pone.0262224.r004

28 Oct 2021

To Reviewer #1

Thank you for your careful comments. The corrections are indicated by red-font text in the revised manuscript.

1) In the structured Abstract, the authors referred to hyperphosphorylated tau (p-tau) in the Methods but not in the Results. The p-tau (Thr 231) was significantly reduced in the CCH group. Please discuss this potentially interesting result in the Abstract and text.

- As you recommended, we have provided the results of p-tau in the Abstract. Possible reasons for the discordant results for the p-tau level have also been provided in the Discussion section (page 14, 1st paragraph).

2) The authors found significant increase of CBF in the CCH group at 3 months. Longitudinal measurement of CBF is thought not to be accurate at 1 month and 3 months after surgery. Please discuss this. The authors stated that the gradual recovery of the CBF could be caused by supply from the collateral blood vessels, such as through the posterior communicating artery. This is a plausible explanation of the result shown in Figure 2. However, do collateral blood vessels induce significant increase of the CBF at 3 months? Please discuss the underlying mechanism of the overshoot of compensatory responses.

- We agree with your comment. The Laser Doppler flowmetry (LDF) may not suitable for the longitudinal measurement of CBF due to the limitations of LDF, such as the influence of the optical properties of tissues on the perfusion signal, motion artifact noise, lack of quantitative units for perfusion, and the biological zero signal. In addition to these limitations of LDF, the possible underlying mechanisms of the overshoot of compensatory responses are described in the Discussion section (page 12, 2nd paragraph).

3) An important reference is not included. FDG-PET study in a mouse model of CCH was previously reported in PMID: 20448204. This should be included in the references and should be thoroughly discussed regarding similarities and differences in the findings between mice and rats.

- We agree with your insightful comment. A previous study involving the CCH mouse model, which you mentioned, reported that glucose metabolism in the hippocampus, as well as in the cortex, decreased for up to 6 months after bilateral CCA ligation, failing to prove the selective vulnerability to ischemia in the hippocampus. This differs from our results showing that the hippocampus is more vulnerable to ischemia than the cortex. The following may account for the difference in results: the compensation mechanisms for recovery of CBF did not occur more extensively in mice than in rats, and the decreased CBF in the cortex affected the decrease in cerebral glucose metabolism. The Discussion section has been updated accordingly (page 13, 2nd paragraph). 

To Reviewer #2

Thank you for your careful comments. The corrections are indicated by red-font in the revised manuscript.

1) It is unclear how this study is sufficiently different from the previously published work, except for addition of the 1 month time point of glucose metabolism.

- We agree with your comment. It is important to note the differences between the results of previous studies and the present study. A previous F-18 FDG PET study of a CCH rat model revealed that CCH decreased the glucose metabolism in the hippocampus 3 months after bilateral CCA ligation, inducing AD pathology. Following the previous study, the present study revealed that glucose metabolism in the anterodorsal hippocampus was decreased by CCH 3 months after the ligation. In addition to the previous study, the present study also showed that the decreased glucose metabolism in the cortex 1 month after the ligation improved after 3 months with the recovery of cerebral blood flow; the decrease in glucose metabolism in the hippocampus was observed 3 months after the ligation. These findings support the ischemic hypothesis for AD development related to CCH, showing that the hippocampus is more vulnerable to ischemia than the cortex. The Introduction and Discussion sections have been updated accordingly (page 3, last paragraph and page 13, 2nd paragraph).

2) Sex and age are not appropriately considered ask key biological variables. Only young males are used. Dementia is a disease of aging, so use of young animals should be discussed as a limitation. Further, use of only one sex should be discussed as a major limitation.

- Old age is the strongest risk factor for AD, and the mechanisms for AD development can differ with sex; therefore, evaluating the differences in the effects of chronic cerebral ischemia according to sex in older rats may help elucidate the mechanism of AD development more clearly. This limitation has been added to the Discussion section (page 15, last paragraph).

3) Behavior test results are inaccurately described. Y maze is described as an assessment of learning and recognition memory. However, as tested, the Y maze protocol used assesses working memory. The test should be revised.

- As you mentioned, the Y-maze test can be used to assess short-term working memory in rats. We have revised the manuscript to highlight the characteristics of the Y-maze test in the Methods and Discussion sections (page 14, last paragraph). 

4) Use of only 1 behavior tests with no control tests that assessed anxiety-like behavior or locomotor activity is a limitation.

- We agree that is a limitation of the present study. We have added it to limitation in the Discussion section (page 15, last paragraph).

5) Use of laser Doppler probe for blood flow measurement should be discussed as a limitation. This method allows assessment of only one very small regional of interest and may not reflect a lack of cerebral hypoperfsion in the entire brain at this time point.

- We agree with your comment. The CBF, which was measured by Laser Doppler flowmetry, was for a specific region in the cerebral cortex rather than the entire brain. This is related to the limitation of Laser Doppler flowmetry, with lack of the knowledge of the depth of CBF measurement. We have highlighted this limitation in the Discussion section (page 13, 1st paragraph). 

6) Graphs could be improved. Some are pixelated and none include the individual data points.

- Figures 4, 5, and 6 have been modified to include individual data points.

7) Please mention whether you are assessing soluble or insoluble amyloid beta.

- Western blots were used to assess the expressions of soluble Aβ, Aβ40, and Aβ42. We have described this in the Methods section (page 6, last paragraph).

8) Please add lot numbers for primary antibodies.

- We agree with your comment. It is important to record the lot number, because the same primary antibody may yield different results depending on the factory, date of manufacture, and raw material. Unfortunately, we did not record the lot number. We are sorry for not providing the lot number.

9) Please reference table 1 (in addition to 4A-B) when describing differences in cerebral metabolism.

- As you suggested, we have added Table 1 and Figure 4A-B to provide further details on the differences in cerebral metabolism in the Results section (page 9, 1st paragraph).

Attachment Submitted filename: Response_to_Reviewers_2.1.docx Click here for additional data file.

# Peer review: Decision Letter 2

**DOI:** 10.1371/journal.pone.0262224.r005

**Authors:** Alexander A. Mongin

23 Nov 2021

PONE-D-21-25820R2Selective Neurodegeneration of the Hippocampus Caused by Chronic Cerebral Hypoperfusion: F-18 FDG PET Study in RatsPLOS ONE

Dear Dr. Kim,

Thank you for submitting your manuscript to PLOS ONE. After careful consideration of the revised version, two reviewers and Academic Editor feel that it has merit and is potentially publishable.  However, the reviewers pointed to two additional issues that would have to be addressed.  Therefore, we invite you to submit a revised version of the manuscript that addresses the points raised during the review process.

Specifically:

[1] Reviewer 1 has a valid point about the effects of bilateral CCA ligation in MICE, which are misquoted in your manuscript on p. 13.  Unlike rats, many strains of mice have low patency of the posterior communicators.  The original paper, which tested the effects of bilateral CCA ligations in mice (K Kitawa et al, JCBFM, 1998), identified robust forebrain/hippocampal damage after only 15 min of bilateral CCA ligation, particularly in C56BL/6.  Indeed, I think that the manuscript by Kitawa et al. showing incomplete and variable patency of posterior communicator arteries in mice strains has a place in your discussion.  It highlights the difference in perfusion patterns between species with pathological implications.

[2] Reviewer 2 requested additional clarity on statistical analysis.  Specifically, you need to clarify which test was used for analyzing  differences in western blot immunoreactivities.  These cannot be analyzed using two populations t-test, but rather one-population t-test where values are compared to the unity.  If this is not the case, please re-analyze the data and make appropriate corrections in the text.

Assuming these two requirements are met, your manuscript will be accepted.

Please submit your revised manuscript by Jan 07 2022 11:59PM. If you will need more time than this to complete your revisions, please reply to this message or contact the journal office at plosone@plos.org. When you're ready to submit your revision, log on to https://www.editorialmanager.com/pone/ and select the 'Submissions Needing Revision' folder to locate your manuscript file.

Please include the following items when submitting your revised manuscript:

- A rebuttal letter that responds to each point raised by the academic editor and reviewer(s). You should upload this letter as a separate file labeled 'Response to Reviewers'.
- A marked-up copy of your manuscript that highlights changes made to the original version. You should upload this as a separate file labeled 'Revised Manuscript with Track Changes'.
- An unmarked version of your revised paper without tracked changes. You should upload this as a separate file labeled 'Manuscript'.

If you would like to make changes to your financial disclosure, please include your updated statement in your cover letter. Guidelines for resubmitting your figure files are available below the reviewer comments at the end of this letter.

If applicable, we recommend that you deposit your laboratory protocols in protocols.io to enhance the reproducibility of your results. Protocols.io assigns your protocol its own identifier (DOI) so that it can be cited independently in the future. For instructions see: https://journals.plos.org/plosone/s/submission-guidelines#loc-laboratory-protocols. Additionally, PLOS ONE offers an option for publishing peer-reviewed Lab Protocol articles, which describe protocols hosted on protocols.io. Read more information on sharing protocols at https://plos.org/protocols?utm_medium=editorial-email&utm_source=authorletters&utm_campaign=protocols.

We look forward to receiving your revised manuscript.

Kind regards,

Alexander A. Mongin, Ph.D.

Academic Editor

PLOS ONE

Journal Requirements:

Please review your reference list to ensure that it is complete and correct. If you have cited papers that have been retracted, please include the rationale for doing so in the manuscript text, or remove these references and replace them with relevant current references. Any changes to the reference list should be mentioned in the rebuttal letter that accompanies your revised manuscript. If you need to cite a retracted article, indicate the article’s retracted status in the References list and also include a citation and full reference for the retraction notice.

[Note: HTML markup is below. Please do not edit.]

Reviewers' comments:

Reviewer's Responses to Questions

**Comments to the Author**

1. If the authors have adequately addressed your comments raised in a previous round of review and you feel that this manuscript is now acceptable for publication, you may indicate that here to bypass the “Comments to the Author” section, enter your conflict of interest statement in the “Confidential to Editor” section, and submit your "Accept" recommendation.

Reviewer #1: All comments have been addressed

Reviewer #2: (No Response)

**********

2. Is the manuscript technically sound, and do the data support the conclusions?

The manuscript must describe a technically sound piece of scientific research with data that supports the conclusions. Experiments must have been conducted rigorously, with appropriate controls, replication, and sample sizes. The conclusions must be drawn appropriately based on the data presented. 

Reviewer #1: Yes

Reviewer #2: No

**********

3. Has the statistical analysis been performed appropriately and rigorously? 

Reviewer #1: Yes

Reviewer #2: No

**********

4. Have the authors made all data underlying the findings in their manuscript fully available?

The PLOS Data policy requires authors to make all data underlying the findings described in their manuscript fully available without restriction, with rare exception (please refer to the Data Availability Statement in the manuscript PDF file). The data should be provided as part of the manuscript or its supporting information, or deposited to a public repository. For example, in addition to summary statistics, the data points behind means, medians and variance measures should be available. If there are restrictions on publicly sharing data—e.g. participant privacy or use of data from a third party—those must be specified.

Reviewer #1: Yes

Reviewer #2: Yes

**********

5. Is the manuscript presented in an intelligible fashion and written in standard English?

PLOS ONE does not copyedit accepted manuscripts, so the language in submitted articles must be clear, correct, and unambiguous. Any typographical or grammatical errors should be corrected at revision, so please note any specific errors here.

Reviewer #1: Yes

Reviewer #2: Yes

**********

6. Review Comments to the Author

Please use the space provided to explain your answers to the questions above. You may also include additional comments for the author, including concerns about dual publication, research ethics, or publication ethics. (Please upload your review as an attachment if it exceeds 20,000 characters)

Reviewer #1: I have only minor comments. The authors stated that ‘Another previous F-18 FDG PET study involving a CCH mouse model revealed that glucose metabolism in the hippocampus, as well as in the cortex, decreased for up to 6 months after bilateral CCA ligation, failing to prove the selective vulnerability to the ischemia in the hippocampus [33].’

This sentence is not accurate because a CCH mouse model is generated by bilateral CCA stenosis with microcoils instead of ligation. Therefore, please correct it accordingly. In addition, it would be better to replace ‘failing to prove’ by ‘but did not show’.

Reviewer #2: I appreciate the author's responses to my comments and additions of limitations to the discussion.

Major comment:

In adding individual data points to the graph, I now see additional issues with rigor. The analysis in Figure 5 is inappropriate. There are no data points for the control group because it was all normalized as 1. There should be normalization to the average of the control group so that there is still an ability to plot and see the variation in individual data points within the controls. Please correct this.

**********

7. PLOS authors have the option to publish the peer review history of their article (what does this mean?). If published, this will include your full peer review and any attached files.

If you choose “no”, your identity will remain anonymous but your review may still be made public.

**Do you want your identity to be public for this peer review?** For information about this choice, including consent withdrawal, please see our Privacy Policy.

Reviewer #1: No

Reviewer #2: No

[NOTE: If reviewer comments were submitted as an attachment file, they will be attached to this email and accessible via the submission site. Please log into your account, locate the manuscript record, and check for the action link "View Attachments". If this link does not appear, there are no attachment files.]

While revising your submission, please upload your figure files to the Preflight Analysis and Conversion Engine (PACE) digital diagnostic tool, https://pacev2.apexcovantage.com/. PACE helps ensure that figures meet PLOS requirements. To use PACE, you must first register as a user. Registration is free. Then, login and navigate to the UPLOAD tab, where you will find detailed instructions on how to use the tool. If you encounter any issues or have any questions when using PACE, please email PLOS at figures@plos.org. Please note that Supporting Information files do not need this step.

# Author comment: Author response to Decision Letter 2

**DOI:** 10.1371/journal.pone.0262224.r006

15 Dec 2021

To Reviewer #1

Thank you for your careful comments. The corrections are indicated by red-font text in the revised manuscript.

1) The authors stated that ‘Another previous F-18 FDG PET study involving a CCH mouse model revealed that glucose metabolism in the hippocampus, as well as in the cortex, decreased for up to 6 months after bilateral CCA ligation, failing to prove the selective vulnerability to the ischemia in the hippocampus [33].’ This sentence is not accurate because a CCH mouse model is generated by bilateral CCA stenosis with microcoils instead of ligation. Therefore, please correct it accordingly. In addition, it would be better to replace ‘failing to prove’ by ‘but did not show’.

Response: We agree with your insightful comment. Nishio et al. reported the effects of chronic cerebral hypoperfusion in mice with bilateral common carotid artery stenosis using microcoils. That sentence has been modified as you commented (page 13, 2nd paragraph). 

To Reviewer #2

Thank you for your careful comments. The corrections are indicated by red-font in the revised manuscript.

1) In adding individual data points to the graph, I now see additional issues with rigor. The analysis in Figure 5 is inappropriate. There are no data points for the control group because it was all normalized as 1. There should be normalization to the average of the control group so that there is still an ability to plot and see the variation in individual data points within the controls. Please correct this.

Response: We agree with your insightful comment. The expression levels for Bax, TNF-α, p-tau (Thr231), Aβ, Aβ40 and Aβ42 in the CCH group were normalized to the average of the control group, and were compared with those of the control group using one-sample t-test. The results of western blotting using one-sample t-test did not differ from the previous results, so the p value was slightly modified. The statistical analysis method used for the western blotting results was modified in the statistical analysis section (page 7, last paragraph), and Figure 5 was also modified accordingly.

Attachment Submitted filename: Response_to_Reviewers_3.0.docx Click here for additional data file.

# Editor report: Decision Letter 3

**DOI:** 10.1371/journal.pone.0262224.r007

**Authors:** Alexander A. Mongin

20 Dec 2021

Selective Neurodegeneration of the Hippocampus Caused by Chronic Cerebral Hypoperfusion: F-18 FDG PET Study in Rats

PONE-D-21-25820R3

Dear Dr. Kim,

We’re pleased to inform you that your manuscript has been judged scientifically suitable for publication and will be formally accepted for publication once it meets all outstanding technical requirements.

Within one week, you’ll receive an e-mail detailing the required amendments. When these have been addressed, you’ll receive a formal acceptance letter and your manuscript will be scheduled for publication.

An invoice for payment will follow shortly after the formal acceptance. To ensure an efficient process, please log into Editorial Manager at http://www.editorialmanager.com/pone/, click the 'Update My Information' link at the top of the page, and double check that your user information is up-to-date. If you have any billing related questions, please contact our Author Billing department directly at authorbilling@plos.org.

If your institution or institutions have a press office, please notify them about your upcoming paper to help maximize its impact. If they’ll be preparing press materials, please inform our press team as soon as possible -- no later than 48 hours after receiving the formal acceptance. Your manuscript will remain under strict press embargo until 2 pm Eastern Time on the date of publication. For more information, please contact onepress@plos.org.

Kind regards,

Alexander A. Mongin, Ph.D.

Academic Editor

PLOS ONE

Additional Editor Comments (optional):

Reviewers' comments:

# Editor report: Acceptance letter

**DOI:** 10.1371/journal.pone.0262224.r008

**Authors:** Alexander A. Mongin

2 Feb 2022

PONE-D-21-25820R3 

Selective Neurodegeneration of the Hippocampus Caused by Chronic Cerebral Hypoperfusion: F-18 FDG PET Study in Rats 

Dear Dr. Kim:

I'm pleased to inform you that your manuscript has been deemed suitable for publication in PLOS ONE. Congratulations! Your manuscript is now with our production department. 

If your institution or institutions have a press office, please let them know about your upcoming paper now to help maximize its impact. If they'll be preparing press materials, please inform our press team within the next 48 hours. Your manuscript will remain under strict press embargo until 2 pm Eastern Time on the date of publication. For more information please contact onepress@plos.org.

If we can help with anything else, please email us at plosone@plos.org. 

Thank you for submitting your work to PLOS ONE and supporting open access. 

Kind regards, 

PLOS ONE Editorial Office Staff

on behalf of

Dr. Alexander A. Mongin 

Academic Editor

PLOS ONE
